# PD-L1 blockade immunotherapy rewires cancer-induced emergency myelopoiesis

**DOI:** 10.3389/fimmu.2024.1386838

**Published:** 2024-10-11

**Authors:** Athina Boumpas, Antonis S. Papaioannou, Pavlos Bousounis, Maria Grigoriou, Veronica Bergo, Iosif Papafragkos, Athanasios Tasis, Michael Iskas, Louis Boon, Manousos Makridakis, Antonia Vlachou, Eleni Gavriilaki, Aikaterini Hatzioannou, Ioannis Mitroulis, Eirini Trompouki, Panayotis Verginis

**Affiliations:** ^1^ Laboratory of Immune Regulation and Tolerance, Division of Basic Sciences, Medical School, University of Crete, Heraklion, Greece; ^2^ Clinical, Experimental Surgery and Translational Research, Biomedical Research Foundation Academy of Athens (BRFAA), Athens, Greece; ^3^ Faculty of Biology, University of Freiburg, Freiburg, Germany; ^4^ Department of Cellular and Molecular Immunology, Max Planck Institute of Immunobiology and Epigenetics, Freiburg, Germany; ^5^ First Department of Internal Medicine, University Hospital of Alexandroupolis, Democritus University of Thrace, Alexandroupolis, Greece; ^6^ Department of Cellular and Molecular Immunology, International Max Planck Research School for Molecular and Cellular Biology (IMPRS-MCB), Freiburg, Germany; ^7^ The Institute of Molecular Biology and Biotechnology of the Foundation for Research and Technology Hellas (IMBB-FORTH), Heraklion, Greece; ^8^ Hematology Department, BMT Unit, G Papanicolaou Hospital, Thessaloniki, Greece; ^9^ JJP Biologics, Warsaw, Poland; ^10^ Biotechnology Division, Biomedical Research Foundation, Academy of Athens (BRFAA), Athens, Greece; ^11^ Institute for Clinical Chemistry and Laboratory Medicine, University Hospital and Faculty of Medicine, Technische Universität Dresden, Dresden, Germany; ^12^ IRCAN Institute for Research on Cancer and Aging, INSERM Unité 1081, Centre National de la Recherche Scientifique (CNRS) Unité Mixte de Recherche (UMR), Université Côte, Nice, France

**Keywords:** cancer, immunotherapy, bone marrow, hematopoietic stem and progenitor cell, inflammation

## Abstract

**Introduction:**

Immune checkpoint blockade (ICB) immunotherapy has revolutionized cancer treatment, demonstrating exceptional clinical responses in a wide range of cancers. Despite the success, a significant proportion of patients still fail to respond, highlighting the existence of unappreciated mechanisms of immunotherapy resistance. Delineating such mechanisms is paramount to minimize immunotherapy failures and optimize the clinical benefit.

**Methods:**

In this study, we treated tumour-bearing mice with PD-L1 blockage antibody (aPD-L1) immunotherapy, to investigate its effects on cancer-induced emergency myelopoiesis, focusing on bone marrow (BM) hematopoietic stem and progenitor cells (HSPCs). We examined the impact of aPD-L1 treatment on HSPC quiescence, proliferation, transcriptomic profile, and functionality.

**Results:**

Herein, we reveal that aPD-L1 in tumour-bearing mice targets the HSPCs in the BM, mediating their exit from quiescence and promoting their proliferation. Notably, disruption of the PDL1/PD1 axis induces transcriptomic reprogramming in HSPCs, observed in both individuals with Hodgkin lymphoma (HL) and tumour-bearing mice, shifting towards an inflammatory state. Furthermore, HSPCs from aPDL1-treated mice demonstrated resistance to cancer-induced emergency myelopoiesis, evidenced by a lower generation of MDSCs compared to control-treated mice.

**Discussion:**

Our findings shed light on unrecognized mechanisms of action of ICB immunotherapy in cancer, which involves targeting of BM-driven HSPCs and reprogramming of cancer-induced emergency myelopoiesis.

## Introduction

Immune checkpoint blockade (ICB) immunotherapy has revolutionized cancer treatment, offering major therapeutic advantages across various cancer types as well as durable clinical responses in cancer patients (1, 2). To date, ICB immunotherapy targets the programmed cell death 1 (PD-1) and its ligand (PD-L1) (3), as well as cytotoxic T lymphocyte antigen 4 (CTLA-4) (4), which mediate dominant immunosuppressive signals. Despite the enormous success, the overall response rates to ICB immunotherapy remain low, highlighting the existence of unappreciated mechanisms of immune checkpoint resistance (1). Importantly, patients that respond to ICB often develop life-threatening immune-related adverse events (irAEs), which present a significant drawback in clinical applications (5, 6). There is an urgent need to comprehensively discern the mechanisms of action of ICB to optimize their therapeutic efficacy and minimize adverse events. Among the three ICB targets, the PD-L1 receptor has gained particular interest since it is broadly expressed by host and cancer cells. To this end, PD-L1 is expressed by myeloid cells such as dendritic cells (DCs), myeloid-derived suppressor cells (MDSCs) and macrophages, activated T and B lymphocytes, and fibroblasts and by certain epithelial cells upon inflammatory signaling (7). Notably, tumor cells also express various levels of PD-L1, which is considered an immune escape mechanism (8). Although it is evident that the expression of PD-L1 by immune cells and tumor cells is required to promote tumor immune evasion and growth (9), still the precise mechanisms through which PD-L1 targeting contributes to the development of antitumor immunity remain poorly understood. Previous studies have shown that aPD-L1 can reinvigorate exhausted CD8^+^ T cells (10) and facilitate the *de-novo* priming of cytotoxic responses in the tumor-draining lymph nodes by interfering with the antigen presentation capacity of DCs (11). Furthermore, PD-L1 blockade of human DCs induced the activation of caspase-1/NLRP3 inflammasome and the release of inflammasome-dependent cytokines (12). Also, PD-L1 blockade triggered an inflammatory signature in mouse macrophages *in vivo* and *in vitro*, promoting antitumor immunity (13). Interestingly, treating asymptomatic multiple myeloma (AMM) patients with atezolizumab, the humanized IgG1 monoclonal antibody targeting PD-L1 (12), induced an inflammatory signature in CD14^+^ monocytes (12), proposing that the PD-L1 axis may shape the myeloid-mediated inflammatory responses. In line with this hypothesis, myeloid skewing has been also implicated in resistance to αPD-L1 immunotherapy. For example, the neutrophil-to-lymphocyte ratio (NLR) in the periphery of lymphoma patients has been proposed to correlate with the lack of response to αPD-L1 treatment (14). Similarly, in advanced non-small cell lung cancer patients, responses to aPD-L1 were associated with decreased frequencies of regulatory T cells (Tregs) (15, 16) and MDSCs as well as a reduction in NLR after treatment (16). However, whether αPD-L1 immunotherapy imprints on the emergency myelopoiesis during cancer to alter the myeloid output in the periphery or directly modulates mature myeloid cell pool remains to be investigated. In addition, to what extent αPD-L1 immunotherapy alters cancer-related myelopoiesis to promote cancer regression while fueling autoimmune adverse events remains elusive. Addressing these unanswered questions will provide important insights toward the design of rational therapies aiming to overcome αPD-L1 resistance and to limit undesired systemic events.

Herein, we demonstrate that αPD-L1 immunotherapy targets the hematopoietic stem and progenitor cells (HSPCs) in the bone marrow (BM), regulating the cancer-induced emergency myelopoiesis. Specifically, αPD-L1 treatment increases the frequencies of BM-derived HSPCs and promotes their exit from quiescence in mice inoculated with either immunogenic or non-immunogenic tumors. Importantly, transcriptomic analysis showed that blocking the PD-L1/PD-1 axis induces inflammatory reprogramming in HSPCs from mice and individuals with Hodgkin lymphoma (HL). Additionally, transplantation of αPD-L1-treated HPSCs significantly altered the cancer emergency myelopoiesis by reducing the frequencies of peripheral MDSCs. Overall, our findings shed light on the unappreciated mechanisms of ICB immunotherapy, which consist of targeting the BM HSPCs and rewiring cancer emergency myelopoiesis while providing new directions for understanding ICB immunotherapy resistance as well as the development of irAEs.

## Materials and methods

### Experimental model and subject details

#### Human subjects

Five patients diagnosed with HL had their BM samples collected for analysis of PD-L1 expression using flow cytometry before initiating treatment. Additionally, BM samples from two of these patients were utilized for RNA sequencing (RNA-seq) of isolated CD34^+^ cells. These were compared to samples from two additional HL patients who had relapsed and were being treated with nivolumab, a humanized IgG4 monoclonal antibody that targets the programmed death-1 (PD-1) receptor (5). Nivolumab was administered at a dose of 240 mg every 2 weeks, with BM samples collected the day before the scheduled dose. The patients receiving nivolumab were in remission at the time of BM collection.

### Animals

C57BL/6J, *Rag1^−/−^* (C57BL/6J background), NBSGW, and *PD-1^−/−^* mice were purchased from the Jackson Laboratory. The NBSGW humanized mouse strain was maintained as homozygotes (*NOD.Cg-Kit^W-41J^Tyr* ^+^ *Prkdc^scid^Il2rg^tm1Wjl^/ThomJ*). *Foxp3^EGFP^*.KI mice (C57BL/6 background) were kindly provided by A. Rudensky (Memorial Sloan–Kettering Cancer Center). Mice were housed six per cage in a temperature- (21°C–23°C) and humidity-controlled colony room, maintained on a 12-h light/dark cycle (07:00 to 19:00 lights on), with standard food (4RF21, Mucedola Srl, Italy) and water provided *ad libitum* and environmental enrichments. All mice in the animal facility were screened regularly by using a health-monitoring program, in accordance with the Federation of European Laboratory Animal Science Association (FELASA), and were free of pathogens. All mice were maintained in the animal facility of the Biomedical Research Foundation of the Academy of Athens (BRFAA) and the Institute of Molecular Biology and Biotechnology Institute (IMBB). During all the experiments, mice were monitored daily. All mice used in the experiments were female and 8–12 weeks old. The total number of mice analyzed for each experiment is detailed in each figure legend. Littermates of the same genotype were randomly allocated to experimental groups.

### Cell lines and primary cell culture

The B16.F10 melanoma and MB49 bladder cancer cell lines used for the solid tumor induction models were kindly provided by A. Eliopoulos (Medical School, National and Kapodistrian University of Athens, Athens, Greece) and were negative for *Mycoplasma* spp., tested by PCR. B16.F10 and MB49 cancer cells were cultured at 37°C under 5% CO_2_ in RPMI-1640 (GlutaMAX™, Gibco, Waltham, Massachusetts, USA, #61870) and DMEM (Gibco, Waltham, Massachusetts, USA, #11965) medium, respectively, supplemented with 10% heat-inactivated fetal bovine serum (FBS, Gibco, Waltham, Massachusetts, USA, #10270), 100 U/ml of penicillin–streptomycin (10,000 U/ml, Gibco, Waltham, Massachusetts, USA, #15140), and 50 μM of 2-mercaptoethanol (50 mM, Gibco, Waltham, Massachusetts, USA, #31350). Cells were split at 90%–100% confluence. All experiments were performed with early passage (p2–3) cells.

Murine-sorted MDSCs and Teff cells were obtained as described below. They were cultured in RPMI-1640 medium containing 10% heat-inactivated FBS, 100 U/ml of penicillin–streptomycin, and 50 μM of 2-mercaptoethanol.

### Solid tumor induction and *in-vivo* immunotherapy administration protocols

Mice were implanted subcutaneously (s.c.) on the back with 3 × 10^5^ B16.F10 melanoma (17) or 75 × 10^4^ MB49 bladder cancer cells. Cancer cell viability was assessed by Trypan blue exclusion. Mice were then euthanized on the day of tumor development indicated in each experimental setup. Mice that manifested tumor ulceration were excluded from the experimental processes.

For the application of immunotherapy, mice were treated intraperitoneally (i.p.) with anti-PD-L1 (αPD-L1) antibody (aPD-L1; 200 μg per 100 μl; i.p.: clone MIH5), anti-CTLA-4 (100 μg per 100 μl in each mouse i.p.: clone 4F10), anti-PD-1 (αPD-1; 200 μg per 100 μl i.p.: clone RMP1–14). Control mouse cohort was administered i.p. PBS on the same days. Immunotherapy or control treatment was administered every 3 days, starting at day 0 of tumor implantation.

### Tissue dissociation and sample preparation

Lymph nodes and spleen were collected from euthanized mice, and single-cell suspensions were obtained by homogenization of the tissues and filtering through a 40-μm cell strainer (BD Falcon, New Jersey, USA) with ice-cold 5% FBS/PBS. Tibiae, femurs, and hip bones were collected, and BM cell suspension was isolated by flushing out the bones with ice-cold 5% FBS/PBS. Red blood cells in the spleen and BM cell suspensions were lysed by incubation in 2 ml of ammonium chloride (NH_4_Cl) for 2 min in RT. Cells from the tumor microenvironment (TME) were isolated by dissociating tumor tissue in the presence of RPMI-1640 (GlutaMAX™, Gibco, Waltham, Massachusetts, USA, #61870) supplemented with collagenase D (1 mg ml^−1^, Roche, Basel, Switzerland) and DNase I (0.25 mg ml^−1^, Sigma, Darmstadt, Germany) for 45 min before passing through a 40-μm cell strainer (BD Falcon, New Jersey, USA). Peripheral blood collection was obtained through the submandibular vein using a 25-gauge needle. To prevent blood from clotting, a solution of 0.1 M EDTA was used for coating syringes, needles, and tubes. PBMCs were isolated on Lymphocyte Separation Media (Lymphosep; Biowest, Nuaillé, France, #L0560). Tubes were centrifuged at 500*g* for 30 min with no brake RT. The PBMC layer was collected, and cells were washed with PBS.

Human BM aspirates were collected from patients with HL, and BM mononuclear cells were isolated by density gradient centrifugation, using Ficoll-Histopaque 1077 (Sigma-Aldrich, Darmstadt, Germany, #10771).

### Flow cytometry and cell sorting

For extracellular marker staining, single-cell suspensions from murine tumor, spleen, LNs, peripheral blood, or BM were incubated for 20 min at 4°C with the following antimouse conjugated antibodies: anti-CD45-PerCP/Cy5.5 (BioLegend, San Diego, California, USA, clone 30-F11, #103132), anti-CD45.1-PE/Cyanine7 (BD Biosciences, San Jose, California, USA, clone A20, #560578), anti-CD11c-APC (BioLegend, San Diego, California, USA, clone N418, #117310), anti-CD11c-FITC (BioLegend, San Diego, California, USA, clone N418, #117306), anti-CD11c-PE (BioLegend, San Diego, California, USA, clone N419, #117308), anti-CD11b-PE/Cyanine7 (BioLegend, San Diego, California, USA, clone M1/70, #101216), anti-CD11b-Brilliant Violet 510 (BioLegend, San Diego, California, USA, clone M1/70, #101263), anti-CD11b-FITC (BioLegend, San Diego, California, USA, clone M1/70, #101206), anti-Gr-1-PE (BioLegend, San Diego, California, USA, clone RB6-8C5, #108408), anti-Gr-1-PE/Cyanine7 (BioLegend, San Diego, California, USA, clone RB6-8C5, #108416), anti-Gr-1-FITC (BioLegend, San Diego, California, USA, clone RB6-8C5, #108406), anti-Ly-6G-PE (BioLegend, San Diego, California, USA, clone 1A8, #127608), anti-Ly-6G-PE/Cyanine7 (BioLegend, San Diego, California, USA, clone 1A8, #127618), anti-Ly-6C-Brilliant Violet 421 (BioLegend, San Diego, California, USA, clone HK1.4, #128032), anti-Ly-6C-PerCP (BioLegend: San Diego, California, USA, clone HK1.4, #128028), anti-CD274 (B7-H1, PD-L1)-Brilliant Violet 421 (BioLegend, San Diego, California, USA, clone 10F.9G2, #124315), anti-CD274 (B7-H1, PD-L1)-Brilliant Violet 421 (BD Pharmingen, clone MIH5, #564716), anti-CD274 (B7-H1, PD-L1)-PE/Dazzle (BioLegend, San Diego, California, USA, clone 10F.9G2, #124323), anti-IgG2a,λ-BV421 (BD, clone B39-4, #562965), anti-TER-119-FITC (BioLegend, San Diego, California, USA, clone TER-119, #116206), anti-CD45R/B220-FITC (BioLegend, San Diego, California, USA, clone RA3-6B2, #103206), anti-CD16/32-FITC (BioLegend, San Diego, California, USA, clone 93, #101306), anti-CD16/32-PE/Cyanine7 (BioLegend, San Diego, California, USA, clone 93, #101317), anti-CD117 (c-Kit)-PE (BioLegend, San Diego, California, USA, clone 2B8, #105808), anti-Ly-6A/E (Sca-1)-APC (BioLegend, San Diego, California, USA, clone E13-161.7, #122512), anti-Ly-6A/E (Sca-1)-Brilliant Violet 421 (BioLegend, San Diego, California, USA, clone D7, #108127), anti-CD48-Alexa Fluor 700 (BioLegend, San Diego, California, USA, clone HM48-1, #103426), anti-CD150-PE/Cyanine7 (BioLegend, San Diego, California, USA, clone TC15-12F12.2, #115914), anti-CD34-Brilliant Violet 421 (BioLegend, San Diego, California, USA, MEC14.7, #119321), anti-CD135-Brilliant Violet 421 (BioLegend, San Diego, California, USA, clone A2F10, #135313), anti-CD127 (IL-7Rα)-PerCP/Cyanine5.5 (BioLegend, San Diego, California, USA, clone SB/199, #121114), anti-CD3ϵ-Pacific Blue (BioLegend, San Diego, California, USA, clone 145-2C11, #100334), and anti-CD4-Brilliant Violet 510 (BioLegend, San Diego, California, USA, clone GK1.5, #100449). Fluorescence minus one (FMO) and isotype (**Supplementary Figure 3A**) were used as a negative control, to increase the accuracy of gate placement. Data acquisition was performed on FACSAria III (BD Biosciences, San Jose, California, USA), FACSCelesta (BD Biosciences, San Jose, California, USA), FACS Canto II (BD Biosciences, San Jose, California, USA), and BD FACSDiva v8.0.1 software (BD Biosciences, San Jose, California, USA). Murine splenic MDSCs, T effector cells, and BM HSPCs were sorted on a FACSAria III v8.0.1 software (BD Biosciences, San Jose, California, USA). Cell purity was above 95%. Flow cytometry data were analyzed with FlowJo v.8.7 and 10.8.1 software. The percentages of the presented cell types were calculated relative to the total cell count obtained through flow cytometry.

Human BM mononuclear cells were stained for extracellular surface markers in a staining buffer (2% FBS/PBS) for 20 min at 4°C before acquisition via flow cytometry. The following human monoclonal antibodies were used: anti-CD34-FITC (BioLegend, San Diego, California, USA, clone 581, #343504), anti-CD34-APC (BD Biosciences, San Jose, California, USA, clone 8G12, #345804), anti-PD-L1-PE (BioLegend, San Diego, California, USA, clone 29E.2A3, #329705), anti-CD45-PerCP (BD Biosciences, San Jose, California, USA, clone 2D1, #347464), and anti-CD38-APC-H7 (BD Biosciences, San Jose, California, USA, clone HB7, #653314). FMO was used as a negative control to increase the accuracy of gate placement. Cell acquisition was performed with a FACS Canto II flow cytometer (BD Biosciences, San Jose, California, USA), and cells were sorted on a FACSAria III v8.0.1 software (BD Biosciences, San Jose, California, USA). Cell purity was above 95%.

### Cell cycle assessment

For the cell cycle analysis via flow cytometry, 10^6^ BM HSPCs (Lin^−^Sca1^+^cKit^+^) per sample were first stained extracellularly as previously described, fixed and permeabilized using fixation/permeabilization buffer (Foxp3/TF Buffer Set; eBioscience, Waltham, Massachusetts, USA, #00552300), and subsequently stained with Ki-67-PE/Cyanine7 (BioLegend, San Diego, California, USA, clone 16A8, #652425).

### HSPC transplantation

CD45.2^+^-C57BL/6 mice were inoculated with B16.F10 melanoma cells and treated with αPD-L1 or PBS as previously described. Eight days after injection, mice were euthanized, BM cells were isolated as previously described, and 2 × 10^4^ HSPCs (Lin^−^Sca1^+^cKit^+^) were injected in the orbital vein of humanized mice CD45.1^+^-NBSGW. Six to 7 weeks post-injection, NBSGW mice were either sacrificed or subcutaneously injected with B16.F10, as previously described. After 17 days, NBSGW mice were euthanized, and the lineage output was measured through flow cytometry.

### *In-vitro* suppression assay

For the suppression assay of MDSC subsets, CD4^+^Foxp3^−^ effector T cells (Teff) were sorted from the LNs of naive Foxp3^EGFP^.KI mice as described previously (18, 19) and stained with the division-tracking dye CellTrace carboxyfluorescein diacetate succinimidyl ester (CFSE) (Invitrogen, Waltham, Massachusetts, USA, #C34554) according to the manufacturer’s protocol. In summary, a total of 75 × 10^3^ labeled Teff cells were seeded in a 96-well round-bottom plate in each well supplemented with Dynabeads™ Mouse T-Activator CD3/CD28 (Gibco, Waltham, Massachusetts, USA, #11456D) at a ratio of 1:1 beads to Teff cells. M-MDSC (CD11b^high^Ly6C^+^Ly6G^–^) and G-MDSC (CD11b^high^Ly6C^–^Ly6G^+^) subsets, sorted from the spleens of C57BL/6J B16.F10-inoculated mice treated with either αPD-L1 or control (PBS), were added to the culture for a total of 64 h at a ratio of Teff/M-MDSCs 1:1 and Teff/G-MDSCs 3:1.

### BM fluid isolation and preparation for proteomic analysis

Femurs were isolated and flushed with ice-cold PBS in Eppendorf tubes. The BM supernatant was harvested after pelleting cells by centrifugation at 1,800 rpm for 10 min at 4°C. Cytokine profile was evaluated via mass spectrometry.

Bone marrow supernatants (0.5 ml per sample) were concentrated with 3 kDa MWCO Amicon Ultra Centrifugal filter devices (Merck Millipore, Now part of Merck Group, headquartered in Darmstadt, Germany) up to a final volume of 30 μl. Protease inhibitors were added to the samples and the protein concentration was defined with Bradford assay. Concentrated samples were processed with the filter-aided sample preparation (FASP) method as described previously (20), with minor modifications (21). Briefly, sample volume corresponding to 200 μg of total protein content was mixed with lysis buffer (0.1 M of Tris–HCl pH 7.6, supplemented with 4% SDS and 0.1 M of DTE), and buffer exchange was performed in Amicon Ultra Centrifugal filter devices (0.5 ml, 30 kDa MWCO; Merck Millipore, Now part of Merck Group, headquartered in Darmstadt, Germany) at 14,000 rcf for 15 min at RT. Each sample was diluted with urea buffer (8 M of urea in 0.1 M of Tris–HCl pH 8.5) and centrifuged. The concentrate was diluted again with urea buffer and centrifugation was repeated. Alkylation of proteins was performed with 0.05 M of iodoacetamide in urea buffer for 20 min in the dark at RT, followed by centrifugation at 14,000 rcf for 10 min at RT. Additional series of washes were conducted with urea buffer (two times) and ammonium bicarbonate buffer (50 mM of NH_4_ HCO_3_ pH 8.5, two times). Tryptic digestion was performed overnight at RT in the dark, using a trypsin-to-protein ratio of 1:100. Peptides were eluted by centrifugation at 14,000 rcf for 10 min, lyophilized, and stored at −80°C until further use.

### LC-MS/MS analysis

Samples were resuspended in 200 μl of mobile phase A (0.1% formic acid). A 5-μl volume was injected into a Dionex Ultimate 3000 RSLS nano flow system (Dionex, Camberley, UK) configured with a Dionex 0.1 × 20 mm, 5 μm, 100 Å C18 nano trap column with a flow rate of 5 µl/min. The analytical column was an Acclaim PepMap C18 nano column 75 μm × 50 cm, 2 μm 100 Å with a flow rate of 300 nl/min. The trap and analytical columns were maintained at 35°C. Mobile phase B was 100% acetonitrile:0.1% formic acid. The column was washed and re-equilibrated prior to each sample injection. The eluent was ionized using a Proxeon nano spray ESI source operating in positive ion mode. For mass spectrometry analysis, a Q Exactive Orbitrap (Thermo Finnigan, Bremen, Germany) was operated in MS/MS mode. The peptides were eluted under a 120-min gradient from 2% (B) to 80% (B). Gaseous phase transition of the separated peptides was achieved with positive ion electrospray ionization applying a voltage of 2.5 kV. For every MS survey scan, the top 10 most abundant multiply charged precursor ions between *m*/*z* ratio 300 and 2,200 and intensity threshold 500 counts were selected with FT mass resolution of 70,000 and subjected to HCD fragmentation. Tandem mass spectra were acquired with an FT resolution of 35,000. Normalized collision energy was set to 33 and already targeted precursors were dynamically excluded for further isolation and activation for 15 s with 5 ppm mass tolerance.

### MS data processing

Raw files were analyzed with the Proteome Discoverer 1.4 software package (Thermo Finnigan, Waltham, Massachusetts, USA), using the Sequest search engine and the UniProt mouse (*Mus musculus*) reviewed database, downloaded on 22 November 2017, including 16,935 entries. The search was performed using carbamidomethylation of cysteine as static and oxidation of methionine as dynamic modifications. Two missed cleavage sites, a precursor mass tolerance of 10 ppm, and a fragment mass tolerance of 0.05 Da were allowed. False discovery rate (FDR) validation was based on *q*-value: target FDR (strict): 0.01 and target FDR (relaxed): 0.05.

Normalized serum protein concentrations were imported into R, and sample mean fluorescence intensities were scaled to each other, log2-transformed, and plotted in a heatmap using the heatmap.2 function from the gplots package v3.1.1. Proteins were considered differentially abundant at a cutoff of |FC| ≥1.5 and significant at *p <*0.05, as determined by unpaired two-tailed Student’s *t*-test. Functional enrichment analysis tables of significant differentially abundant proteins were produced with Metascape v3.5 (http://metascape.org), and top hits were visualized in a dot plot using the R ggplot2 package v3.4.1. Gene set enrichment analysis (GSEA v4.2.2 [build: 8]) was performed to reveal enriched signatures in our gene sets based on the Molecular Signatures Database (MSigDB, v7.4; the selected libraries used are represented in **Supplementary Table 4** “GSEA_libraries_MURINE_HUMAN_RNAseq”). Gene sets were ranked by taking the –log10 transform of the *p*-value and multiplying it by the corresponding FC, with significantly upregulated genes at the top of the ranked list. GSEA pre-ranked analysis was performed using the remapped Mouse Gene Symbol dataset and collapsing probe sets while keeping only the max probe value. The rest of the parameters were left to default. Enrichment was considered significant if FDR (*q*-value) <25%. Pathway analysis was performed using tissue-specific Ingenuity Pathway Analysis [IPA Winter Release (Dec 2022), RRID: SCR_008653].

### RNA sequencing library preparation

BM murine HSPCs (Lin^−^Sca1^+^cKit^+^) samples were isolated from B16.F10 melanoma, and MB49-bearing mice were treated with PBS or αPD-L1. Human BM samples, as previously described, were from HL patients isolated at diagnosis or after αPD-1 treatment. HSPCs were sorted and total RNA was extracted using the Arcturus™ PicoPure™ RNA Isolation Kit (Thermo Fisher Scientific, Waltham, Massachusetts, USA, #12204-01).

HSPCs (Lin^−^Sca1^+^cKit^+^) from B16.F10-bearing mice and human BM-derived CD34^+^ cells RNA-seq experiments were carried out at the Greek Genome Center (GGC) of the Biomedical Research Foundation of the Academy of Athens (BRFAA). RNA-seq libraries were prepared with the NEBNext Ultra II Directional RNA Library Prep Kit (Illumina, San Diego, California, USA). Quality control was performed with the Agilent bioanalyzer DNA1000 kit, and quantitation was performed with the Qubit HS spectrophotometric method. Approximately 25 million 100-bp single-end (murine samples) and paired-end (human samples) reads were generated for each sample in the Illumina NovaSeq 6000 system.

RNA-seq library preparation of HSPCs (Lin−Sca1+cKit+) from MB49-bearing mice was carried out at the Max Planck Institute of Immunobiology and Epigenetics (MPI-IE). cDNA libraries were prepared using SMART-seq^®^ v4 Ultra Low Input RNA Kit (#634888, TaKaRa). The NEB Ultra II FS DNA kit (#E7805S) was used to generate barcoded sequencing libraries. Quality control was performed with Agilent 5200 Fragment Analyzer. Fifty million paired-end 101-bp reads per sample were generated using the Illumina HiSeq 3000 or NovaSeq 6000 system at the DeepSequencing Facility at MPI-IE.

### RNA sequencing data processing

RNA-seq data were analyzed and paired-end fastq read files were pre-processed by assessing for quality with FastQC v0.11.9 and trimming off Illumina sequencing adapters with galore Trim Galore v0.3.7. Alignment to the reference mouse genome (GENCODE GRCm38.p6_M230) was carried out with STAR v2.7.10a using the default parameters. HTseq-count v0.12.4 was used to produce gene count matrices from the resulting alignments with the specific parameters –*intersection-non-empty* and additionally *–stranded=“reverse”* (using the reference GENCODE GRCm38.p6_M23 annotation) for RNA libraries prepared with the NEB directional kit. Sample hierarchical clustering and PCA, TMM normalization, scaling, and differential expression analysis via the exact test were performed in R using edgeR v3.34.1. Mouse genes were considered significantly differentially expressed if they met |FC| ≥1.5 and FDR <0.05. Human differentially expressed genes (DEGs) were considered significant at *p*-value <0.05.

Functional enrichment analysis tables of DEGs were produced with g:Profiler version *e109_eg56_p17_1d3191d* web-server, and top hits were visualized as dot plots using the R ggplot2 package v3.4.1. GSEA pre-ranked analysis and IPA were performed on the murine datasets, as described in proteomics.

### Data analysis and statistics

Data are presented as mean ± standard deviation (SD), and bar graphs represent the mean and SD between biologically independent mouse samples or technical replicates, as indicated in corresponding the figure legend. For statistical analysis, all data were analyzed using Prism 8 (GraphPad Software, Inc., La Jolla, USA). Data were analyzed using the two-tailed, parametric, unpaired Student’s *t*-test or the two-tailed, non-parametric Mann–Whitney test, as appropriate after testing for normality of the values with the *F*-test, with 95% confidence intervals. For multiple-group comparisons, one-way and two-way ANOVA and two-way ANOVA Tukey’s were performed. A *p*-value <0.05 was considered to be statistically significant for each dataset.

### Study approval

The study was approved by the Institutional Review Board and Ethics Committee of G. Papanicolaou Hospital (135/2020). All patients gave written informed consent. The study was conducted in compliance with the Helsinki Declaration.

All mice were maintained in the animal facility of the BRFAA and IMBB. All procedures were in accordance with institutional guidelines and were approved by the Institutional Committee of Protocol Evaluation of the BRFAA and the Institutional Committee of Protocol Evaluation of the IMBB together with the Directorate of Agriculture and Veterinary Policy, Region of Attika, Greece (Athens, Greece 299868, 7/4/2022, and 557279, 30/07/2020), and the Directorates of Agricultural Economy and Veterinary, Region of Crete, Greece (Heraklion, Greece, 216160, 20/07/2022).

### Data availability

Human and mouse data are currently under submission: human RNA-seq data in European Genome-Phenome Archive EGA (https://ega-archive.org/), mouse RNA-seq in GEO (https://www.ncbi.nlm.nih.gov/geo/), and mass spectrometry proteomic data in the ProteomeXchange Consortium via the PRIDE (https://www.proteomexchange.org/).

## Results

### Immunotherapy with αPD-L1 contracts the MDSC compartment in tumor-bearing mice

**Figure 1:**
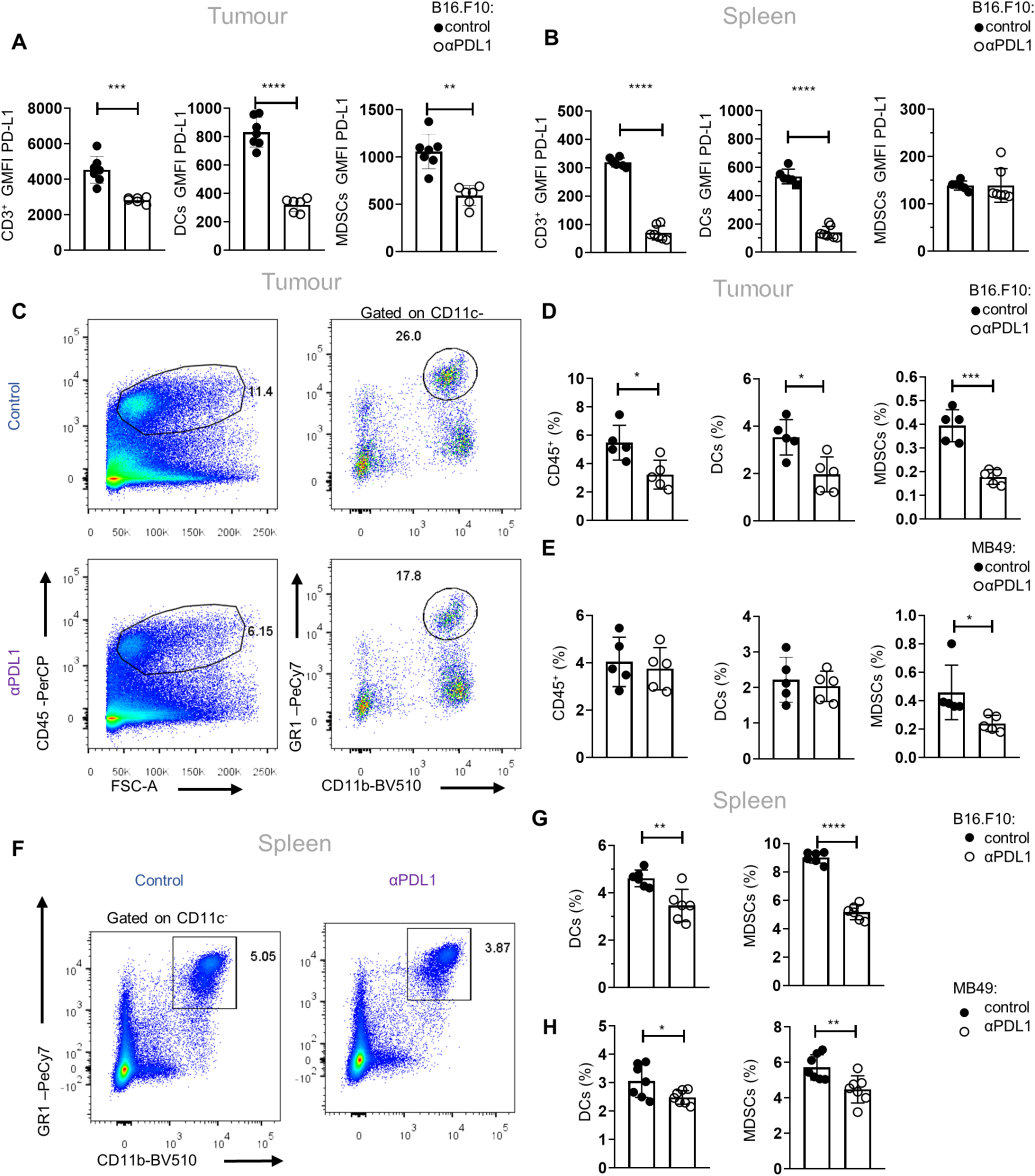
αPD-L1 immunotherapy reduces peripheral MDSC frequencies during tumor progression. **(A, B)** Quantification through flow cytometry of the GMFI of PD-L1 surface expression measurements of intratumoral (**A**; *n* = 7 control, *n* = 6 αPD-L1) and splenic (**B**; *n* = 6 control, *n* = 7 αPD-L1) CD3^+^ cells, CD11c^+^ DCs, and CD11c^−^CD11b^+^Gr1^+^ MDSCs after 8 days in B16.F10 melanoma-bearing C57BL/6 mice treated either with PBS or αPD-L1. Representative data from four independent experiments. **(C–E)** Representative FACS plots (**C**; numbers denote the percentages of gated populations) and quantification of the frequencies in total cells **(D)** of intratumoral CD45^+^ cells, DCs, and MDSCs in PBS- or αPD-L1-treated C57BL/6 mice after 8 days of B16.F10 (**C**, **D**; *n* = 5 control, *n* = 5 αPD-L1) and 12 days of MB49 (**E**; *n* = 5 control, *n* = 5 αPD-L1) tumor progression. Data from one experiment **(D, E)**. **(F–H)** Representative FACS plots (**F**; numbers denote the percentages of gated populations) and frequencies in total cells of splenic DCs and MDSCs during the 8th day of B16.F10 (**F**, **G**; *n* = 6 control, *n* = 6 αPD-L1) and MB49 (**H**; *n* = 7 control, *n* = 7 αPD-L1) tumor progression in C57BL/6 mice treated with PBS or αPD-L1. Data from two independent experiments **(G)** and data from two combined independent experiments **(H)**. *p* < 0.05*, *p* < 0.01**, *p* < 0.001***, *p* < 0.0001****. If not stated otherwise, unpaired two-tailed *t*-tests were performed. Means and SEM are depicted in all bar plots. *n* = biologically independent mouse samples.

To gain an in-depth understanding of the mechanisms underlying the responses to αPD-L1 immunotherapy, we first analyzed the PD-L1 expression in major immune cell populations upon αPD-L1 treatment (clone MIH5) of tumor-bearing mice. Flow cytometric analysis of mice bearing the non-immunogenic B16.F10-melanoma cell line demonstrated significantly decreased geometric mean fluorescent intensity (GMFI) of PD-L1 surface expression in CD3^+^ τ lymphocytes and CD11c^+^ DCs in both the tumor and spleen as well as on intratumoral CD11c^−^CD11b^+^Gr1^+^ MDSCs, when treated with αPD-L1, compared to control (**Figures 1A, B**). Regarding frequencies, CD3^+^ T cells were not altered in the spleen (data not shown) but were increased in the tumors of αPD-L1-treated melanoma-bearing mice (data not shown). Frequencies (percentage of total cells acquired through flow cytometry) of B16.F10 intratumoral DCs and MDSCs were significantly reduced (**Figures 1C, D**; **Supplementary Figure 1A**) in immunotherapy-treated mice. Similar results were observed in MDSCs from αPD-L1-treated mice bearing the immunogenic MB49 bladder carcinoma cell line, while DCs were not affected by the treatment (**Figure 1E**). Decreased frequency of MDSCs was also evident in the peripheral blood of αPD-L1-treated B16.F10-bearing mice compared to control, while DC frequency remained unaltered (**Supplementary Figures 1B, C**). In the spleen, both MDSCs and DCs decreased in the αPD-L1-treated compared to control-treated animals bearing either melanoma (**Figures 1F, G**; **Supplementary Figure 1D**) or bladder cancer (**Figure 1H**). Further analysis of the splenic myeloid compartment showed that the frequencies of the CD11c^−^CD11b^high^Ly6C^+^Ly6G^−^ monocytic MDSC subset (M-MDSCs) significantly decreased in αPD-L1-treated mice inoculated with B16.F10 (**Supplementary Figures 1E, F**); however, MB49-bearing mice presented no differences (**Supplementary Figure 1G**) nor did the CD11c^−^CD11b^high^Ly6C^−^Ly6G^+^ granulocytic MDSC subset (G-MDSCs) in both tumor models (**Supplementary Figures 1E–G**). Overall, these findings demonstrate a significant contraction of the MDSC compartment in the spleen, blood, and tumor of both immunogenic and non-immunogenic tumor-bearing animals upon αPD-L1 treatment.

To examine whether αPD-L1 treatment could also imprint on the functional properties of MDSCs, we performed an *in-vitro* suppression assay. To this end, M-MDSCs and G-MDSCs were isolated from the spleen of control and αPD-L1-treated melanoma-bearing mice and were co-cultured with CellTrace CFSE-labeled T effector (CD4^+^Foxp3^−^) cells sorted from naive Foxp3^EGFP^ mice in the presence of anti-CD3/anti-CD28 activation beads (**Supplementary Figure 2A**). Both MDSC subsets from αPD-L1-treated mice displayed a sustained suppressive ability compared with the control group, as shown by the similar CellTrace CFSE dilution (**Supplementary Figure 2B**), and decreased T-cell activation based on the CD44 and CD25 expression (data not shown). Collectively, these results demonstrate that αPD-L1 treatment significantly decreases the frequencies of MDSCs in tumor-bearing mice without altering their functional properties.

### Human and mouse BM HSPCs express PD-L1

**Figure 2:**
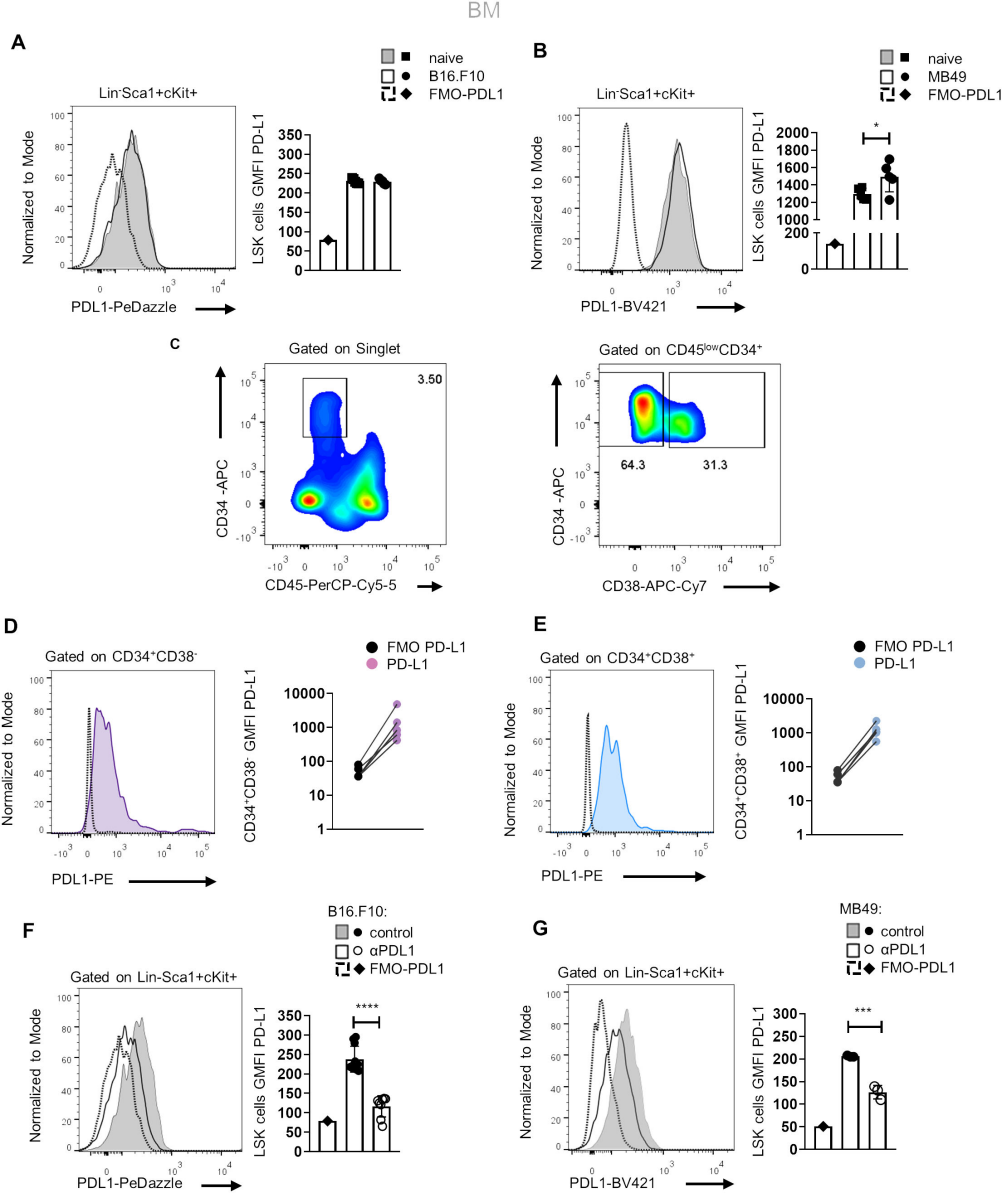
Murine and human HSPCs express PD-L1. **(A, B)** Representative histograms (left) and quantification (right) of surface PD-L1 expression in murine BM HSPCs 8 days following B16.F10 (**A**; *n* = 5 naive, *n* = 5 B16.F10) or MB49 (**B**; *n* = 5 naive, *n* = 5 MB49) inoculation in C57BL/6 mice. Representative flow cytometry data from one **(B)** and two **(A)** independent experiments. **(C–E)** Representative gating strategy (**C**; numbers denote the percentages of gated populations) of human BM stem (CD45^low^CD34^+^CD38^−^) and progenitor (CD45^low^CD34^+^CD38^+^) cells isolated from HL patients at diagnosis. Representative overlays (left) and GMFI quantification (right) of PD-L1 in CD34^+^CD38^−^ (**D**; *n* = 5) and CD34^+^CD38^+^ (**E**; *n* = 5) HL patients compared to their counterpart FMO (representation in a log10 scale). **(F, G)** Representative histograms (left) and quantification (right) of PD-L1 surface staining in BM HSPCs during the 8th day of B16.F10 (**F**; *n* = 8 control, *n* = 11 αPD-L1) or MB49 (**G**; *n* = 3 control, *n* = 3 αPD-L1) tumor development in C57BL/6 mice treated with PBS or αPD-L1. Representative flow cytometry data from 2 **(G)** and 10 (**F**; 2 of them are displayed) independent experiments. *p* < 0.05*, *p* < 0.01**, *p* < 0.001***, *p* < 0.0001****. If not stated otherwise, unpaired two-tailed *t*-tests were performed. Means and SEM are depicted in all bar plots. *n* = biologically independent mouse or human samples.

Since αPD-L1 treatment contracts the MDSC compartment in the periphery of tumor-bearing animals and considering that MDSCs originate from βμ hematopoietic progenitors (22), we hypothesized that αPD-L1 may regulate cancer emergency myelopoiesis. To address this, we first examined whether HSPCs express PD-L1 and if they constitute a target of αPD-L1 immunotherapy. To this end, flow cytometric analysis showed that HSPCs (or LSK cells; lineage (Lin)^−^Sca1^+^cKit^+^) express PD-L1 at steady state and its expression was not altered after B16.F10 melanoma cell inoculation (**Figure 2A**), whereas it was significantly increased upon inoculation with immunogenic MB49 bladder cancer cells (**Figure 2B**). In support, BM stem (CD45^low^CD34^+^CD38^−^, **Figures 2C, D**) and progenitor (CD45^low^CD34^+^CD38^+^, **Figures 2C, E**) cells from patients with HL express PD-L1 as compared to FMO staining.

Notably, treatment with αPD-L1 (clone MIH5) resulted in significantly decreased staining of PD-L1 (clone MIH5 or 10F.9G2) in HSPCs of mice inoculated with either non-immunogenic (**Figure 2F**) or immunogenic tumor cells (**Figure 2G**), pointing to specific targeting of the HSPC compartment. FMO controls and isotype controls were used to ensure the specificity and accuracy of the flow cytometry results (**Figures 2F, G**, **Supplementary Figure 3A**). Together, these results establish that HSPCs in the BM express PD-L1 and are targeted by αPD-L1 immunotherapy in tumor-bearing mice.

### αPD-L1 treatment induces the expansion of HSPCs and their exit from quiescence

**Figure 3:**
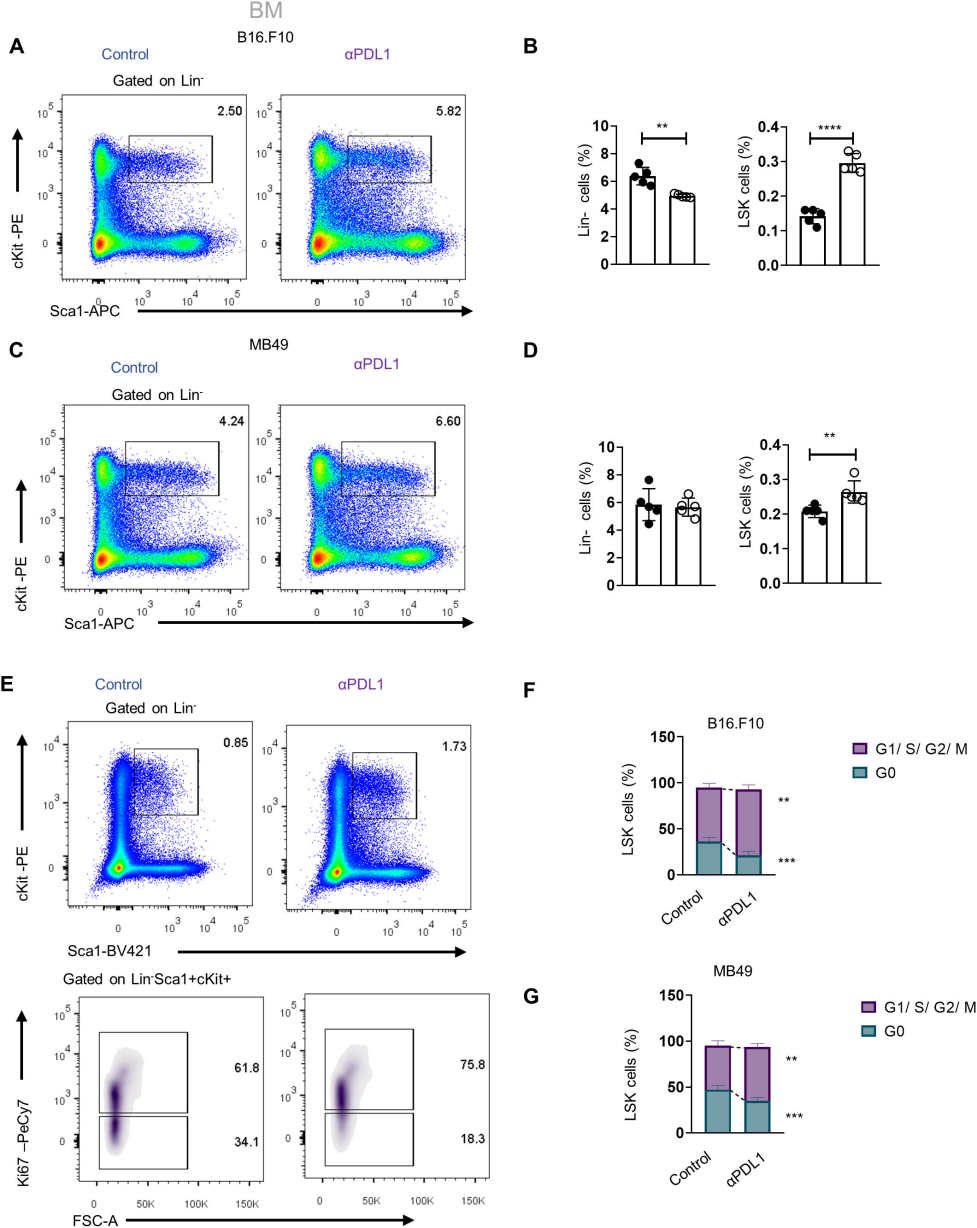
Administration of αPD-L1 drives the expansion of the HSPC compartment and promotes their activation. **(A–D)** Representative FACS plots (**A, C**; numbers denote the percentages of gated populations) and frequencies in total cells of BM HSPCs (LSK: (Lin)^−^Sca1^+^cKit^+^), in PBS- or αPD-L1-treated C57BL/6 mice inoculated with B16.F10 (**B**; **C**; *n* = 5 control, *n* = 5 αPD-L1) and MB49 (**D**; 5 control, *n* = 5 αPD-L1) and sacrificed after 8 days. **(E, F)** Representative FACS plots (**E**; numbers denote the percentages of gated populations) of BM HSPCs isolated from PBS- or αPD-L1-treated C57BL/6 mice inoculated with B16.F10 (**E**, **F**; *n* = 5 control, αPD-L1 *n* = 5) and MB49 (**G**; *n* = 4 control, *n* = 5 αPD-L1). After 8 days, mice were sacrificed and stained with the proliferation marker Ki-67 for cell cycle analysis. Frequencies of HSPCs **(F, G)** in the G0 and G1/S/G2/M cell cycle phases. Two-way ANOVA was performed. Representative data from three (**D**; HSPCs) and nine independent experiments **(B)**. Data from one experiment **(F, G)**. *p* < 0.05*, *p* < 0.01**, *p* < 0.001***, *p* < 0.0001****. If not stated otherwise, unpaired two-tailed *t*-tests were performed. Means and SEM are depicted in all bar plots. *n* = biologically independent mouse samples.

We next asked whether αPD-L1 targeting the HSPCs in the BM affects myelopoiesis. To address this, we first examined if αPD-L1 treatments affect the frequencies of HSPCs during cancer. Notably, αPD-L1 treatment significantly expanded the HSPC compartment in mice inoculated with either non-immunogenic (**Figures 3A, B**; **Supplementary Figure 3B**) or immunogenic tumor cells (**Figures 3C, D**) compared to control-treated mice. Although multipotent progenitors (MPPs) did not demonstrate any differences in αPD-L1-treated B16.F10 mice, the MPP subpopulations with potential for granulocytes and monocytes (MPP^G/M^; LSK^+^Flt3^−^CD48^+^CD150^−^), megakaryocytes and erythrocytes (MPP^Mk/E^; LSK^+^Flt3^−^CD48^+^CD150^+^), and lymphoid cells (MMP^Ly^; LSK^+^Flt3^+^) significantly increased (**Supplementary Figures 3B, C**). A significant increase was also observed in HSCs (LSK^+^Flt3^−^CD48^−^CD150^+^) (**Supplementary Figures 3B, C**). Similar to the results obtained in B16.F10-treated animals, αPD-L1 MB49-bearing injected mice exhibited significantly elevated levels of HSCs and MPPs, with the exception of MMP^Ly^, which were not altered by treatment (**Supplementary Figure 3D**). Collectively, these results demonstrate that αPD-L1 treatment expands the HSPC compartment in the BM of tumor-inoculated animals.

To examine whether αPD-L1 treatment actively induces HSPC proliferation, we performed flow cytometry analysis upon staining with Ki-67 to distinguish proliferating from non-proliferating/quiescent cells. Indeed, αPD-L1-treated B16.F10- (**Figures 3E, F**) and MB49-inoculated (**Figure 3G**) animals showed reduced percentages of HSPCs in the G0 phase and significantly increased percentages in the G1 phase compared to control-treated mice. Taken together, these findings provide evidence that αPD-L1 immunotherapy promotes the exit of HSPCs from the quiescent state and induces their proliferation in the BM.

**Figure 4:**
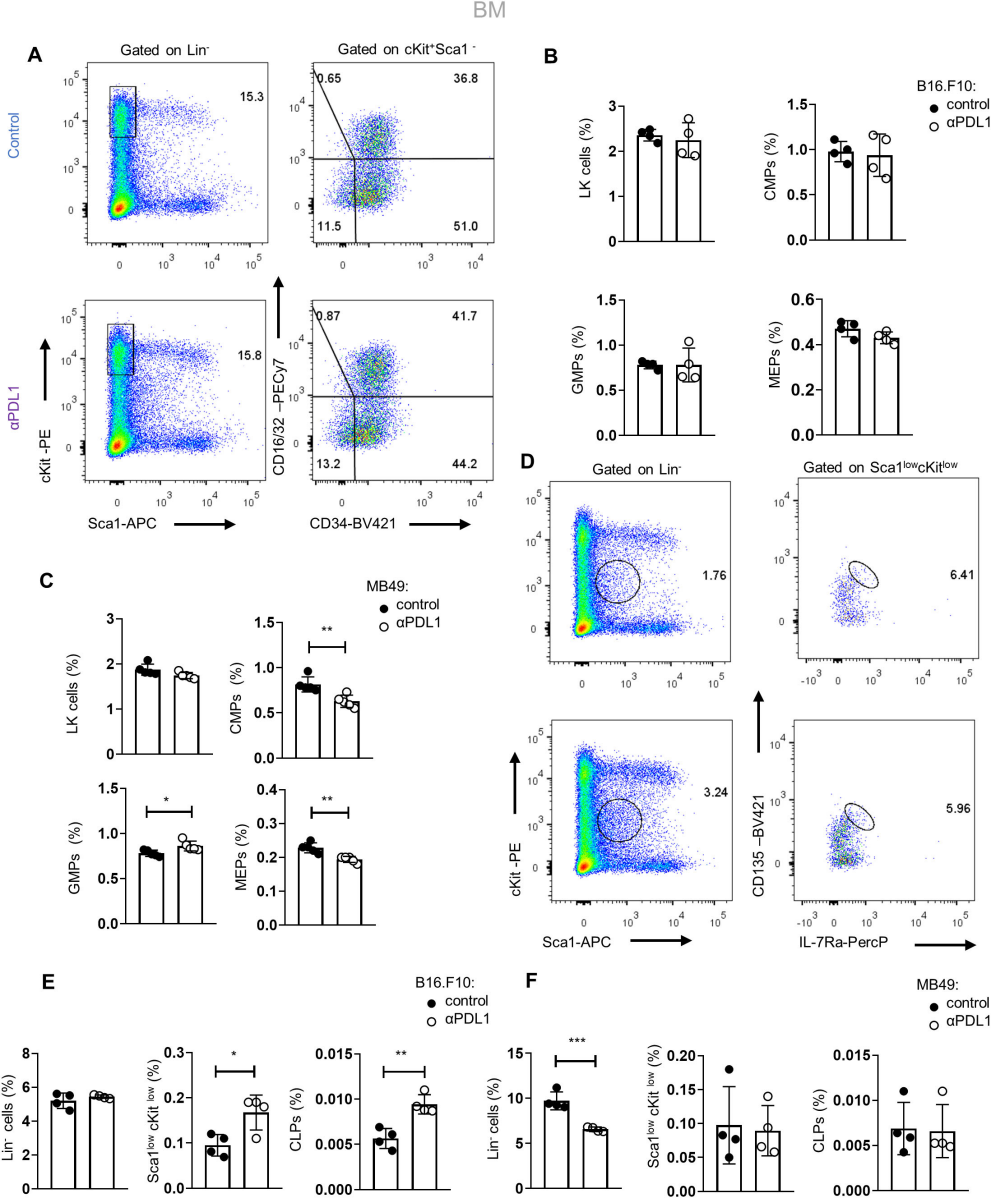
Tumor immunogenicity dictates the differentiation potential of αPD-L1-targeted HSPCs. **(A)** Gating strategy of the BM LK pool (Lin^−^Sca1^−^cKit^+^) and the subclusters CMPs (LK CD34^+^CD16/32^−^), GMPs (LK D34^+^CD16/32^+^), and MEP (LK CD34^−^CD16/32^−^) isolated from PBS- or αPD-L1-treated C57BL/6 mice inoculated with B16.F10 and sacrificed after 8 days. Numbers denote the percentages of gated populations. **(B, C)** Frequencies in total cells of the BM LK compartment, CMPs, GMPs, and MEPs isolated from PBS- or αPD-L1-treated C57BL/6 mice inoculated with melanoma (**B**; *n* = 4 control, *n* = 4 αPD-L1) or MB49 (**C**; *n* = 4 control, *n* = 4 αPD-L1) and sacrificed after 8 days. Representative data from two **(C)** and four **(B)** independent experiments. **(D–F)** Gating strategy of common lymphoid progenitors (CLPs) in the BM (Lin^−^Sca1^low^cKit^low^ IL-7Rα^hi^CD135^hi^) (**D**; numbers denote the percentages of gated populations) and their frequencies in total cells in C57BL/6 mice treated with PBS or αPD-L1 and inoculated with either B16.F10 (**E**; *n* = 4 control, *n* = 4 αPD-L1) or MB49 (**F**; *n* = 4 *n* = 4 control, *n* = 4 αPD-L1) and sacrificed on the 8th day of tumor development. Representative data from one **(F)** and two **(E)** independent experiments. *p* < 0.05*, *p* < 0.01**, *p* < 0.001***, *p* < 0.0001****. Means and SEM are depicted in all bar plots. If not stated otherwise, unpaired two-tailed *t*-tests were performed. *n* = biologically independent mouse samples.

We next asked if αPD-L1 alters the HSPC differentiation potential during the early stages of myeloid commitment. Therefore, we assessed the frequencies of committed myeloid progenitors (LK: Lin^−^Sca1^+^cKit^+^) that can further differentiate into common myeloid progenitors (CMPs; LK CD34^+^CD16/32^−^), granulocyte–macrophage progenitors (GMPs; LK CD34^+^CD16/32^+^), and megakaryocyte–erythrocyte progenitors (MEPs; LK CD34^−^CD16/32^−^). Interestingly, αPD-L1 treatment of non-immunogenic tumor-bearing mice did not affect the frequencies of the myeloid progenitors (**Figures 4A, B**), but the frequency of common lymphoid progenitors (CLPs: Lin^−^Sca1^low^cKit^low^IL7Ra^high^CD135^high^) was significantly increased (**Figures 4D, E**). Contrarily, αPD-L1 treatment decreased the frequency of CMPs and MEPs in immunogenic tumors while increasing the frequency of GMP (**Figure 4C**) without affecting the CLP frequency (**Figure 4F**). Collectively, these findings suggest that αPD-L1 treatment imprints on the expansion of the HSPC compartment, while the immunogenicity of the tumor dictates its differentiation potential.

### Targeting the PD-1/PD-L1 axis expands the HSPC compartment in the BM

**Figure 5:**
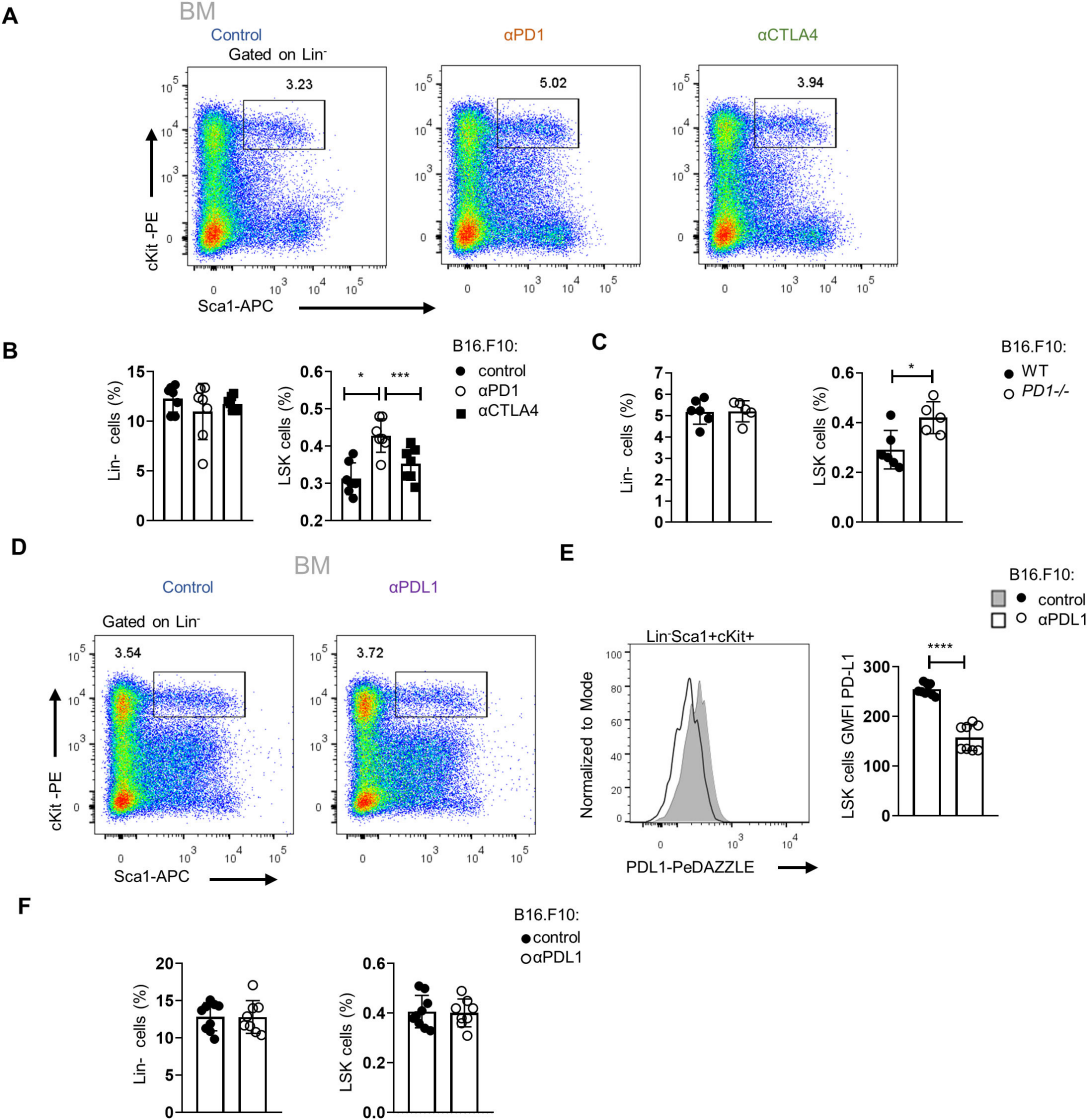
Targeting of the PD-L1/PD-1 axis expands the HSPC compartment in the BM. **(A, B)** Representative FACS plots (**A**; numbers denote the percentages of gated populations) and Lin^−^ cells and HSPC frequencies in total cells (**B**; *n* = 7 control, *n* = 7 αPD-1, *n* = 7 αCTLA-4) in C57BL/6 mice inoculated with B16.F10 and treated with either αPD-1, αCTLA-4, or PBS with BM analysis performed after 8 days. Data from two combined independent experiments. One-way ANOVA was performed. **(C)** Frequencies in total cells of BM Lin^−^ cells and HSPCs, isolated from B16.F10-inoculated *WT* or *PD-1^−/−^* C57BL/6 mice (*n* = 6 WT, *n* = 5 *PD-1^−/−^*), sacrificed during the 8th day of tumor development. **(D–F)** Representative FACS plots (**D**; numbers denote the percentages of gated populations) and frequencies in total cells (**F**; *n* = 9 control, *n* = 8 αPD-L1) of Lin^−^ cells and HSPC subpopulations in *RAG1^−/−^* mice inoculated with B16.F10 and treated with either αPD-L1 or PBS with BM analysis performed after 8 days. Representative histograms (up) and GMFI quantification (down) (**E**; *n* = 9 control, *n* = 8 αPD-L1) of the PD-L1 surface expression of the aforementioned HSPCs. Data from one experiment **(C)** and two combined independent experiments **(B, E, F)** assessed using flow cytometry. *p* < 0.05*, *p* < 0.01**, *p* < 0.001***, *p* < 0.0001****. If not stated otherwise, unpaired two-tailed *t*-tests were performed. Means and SEM are depicted in all bar plots. *n* = biologically independent mouse samples.

To examine whether the expansion of HSPCs in tumor-bearing mice is specific to αPD-L1, we treated mice with either αPD-1 or αCTLA-4, the two ICBs used in the treatment of patients with solid malignancies. Interestingly, only αPD-1 treatment of B16.F10-injected mice demonstrated a significant increase of HSPC frequency, whereas no difference was observed in αCTLA-4-treated mice (**Figures 5A, B**). These results suggest that interfering specifically with the PD-1/PD-L1 axis promotes the expansion of the HSPC compartment. Additionally, a significant increase in HSPC frequencies was observed upon B16.F10 cell inoculation of PD-1-deficient (*PD-1^−/−^*) compared to WT animals (**Figure 5C**; **Supplementary Figures 4A**). Although PD-1 has been shown to be expressed by various cell types of hematopoietic origin (23, 24), T cells constitute the major source of PD-1 expression (23, 25). Therefore, to provide mechanistic insights into our findings, we asked whether PD-1 expression by T cells contributes to the expansion of the HSPC compartment in αPD-L1-treated animals. To this end, *RAG1^−/−^*-immunodeficient animals, which do not harbor T or B lymphocytes due to a defect in the receptor recombination mechanism (26), were inoculated with B16.F10 cells and treated with αPD-L1. Surprisingly, although αPD-L1 efficiently targeted the HSPCs (**Figure 5E**), no significant differences were observed in their frequencies between αPD-L1- and control-treated *RAG1^−/−^* mice (**Figures 5D, F**; **Supplementary Figures 4B, C**). Collectively, these results suggest that targeting the PD-1/PD-L1 axis mediates the expansion of the HSPC compartment during cancer development and highlights an essential role of lymphocytes in this process.

### Enhanced inflammatory signaling and altered myelopoiesis in HSPCs upon αPD-L1 immunotherapy

**Figure 6:**
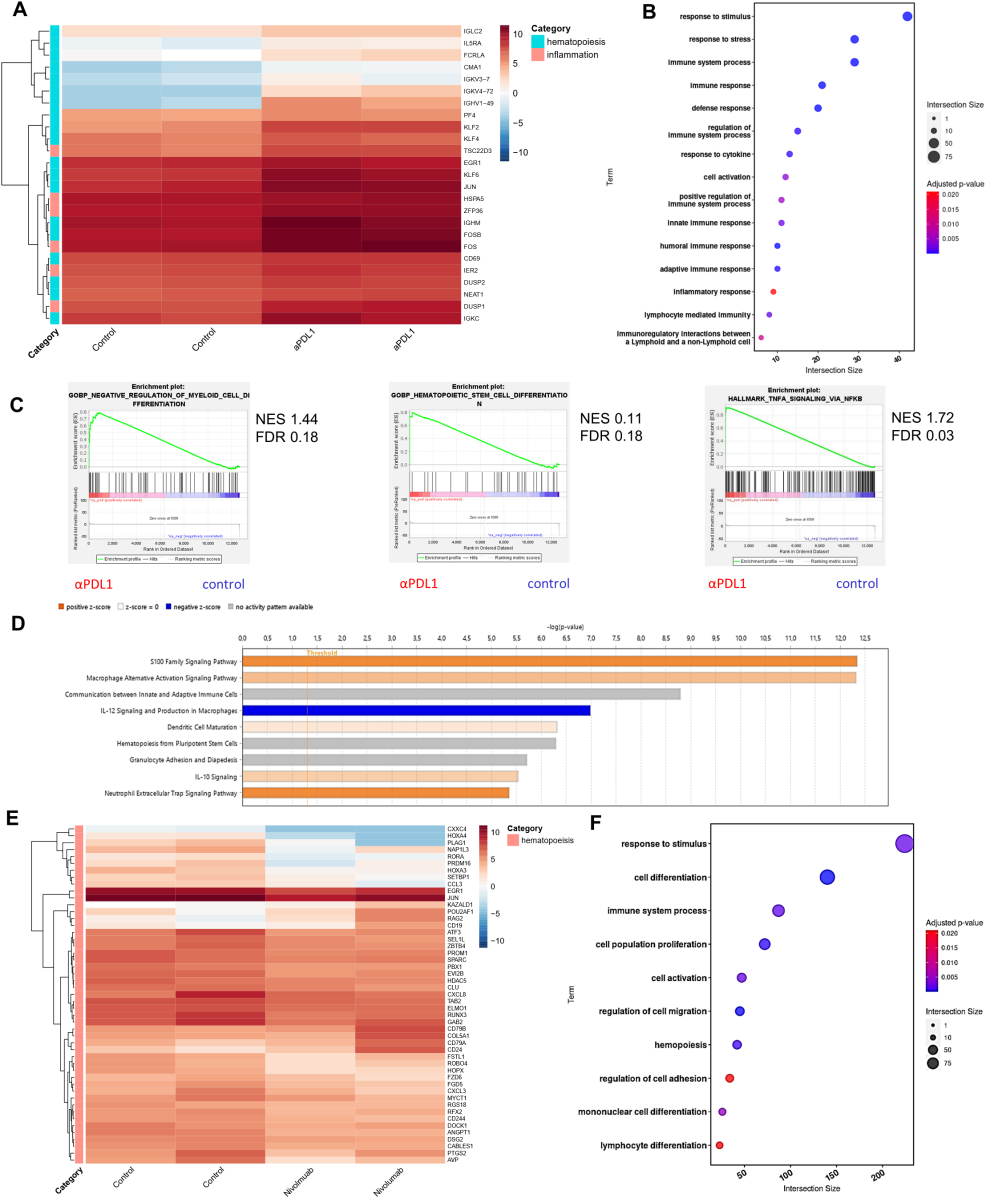
Immunotherapy induces transcriptomic reprogramming of BM HSPCs. **(A)** RNA-seq heatmap of the representative DEGs categorized by their involvement in hematopoiesis and inflammation (|FC| ≥ 1.5, FDR < 0.05) of BM HSPCs isolated from B16.F10 tumor-bearing C57BL/6 mice treated with PBS (control; *n* = 2) or αPD-L1 (*n* = 2). **(B)** Pathway analysis of DEGs from BM HSPCs isolated from B16.F10 tumor-bearing C57BL/6 mice treated with PBS (*n* = 2) or αPD-L1 (*n* = 2). **(C)** GSEA plot showing the positively enriched pathways “negative regulation of myeloid cell differentiation” (NES 1.44, FDR 0.18), “hematopoietic stem cell differentiation” (NES 0.11, FDR 0.18), and “TNF-α signaling via NF-kB” (NES 1.72, FDR 0.03) of the αPD-L1 group compared to control [FDR (*q*-value) < 25%]. **(D)** BM-specific IPA of signaling pathways in BM-HSPCs from melanoma αPD-L1-treated mice, as compared to PBS-treated mice (control). The bar color reflects the IPA activation *z*-score of an enriched pathway which indicates the direction of effect associated from gene to pathway, with orange representing a direct association and blue representing an indirect association between pathway activation/inhibition and gene expression. **(E)** RNA-seq heatmap of representative DEGs involved in hematopoiesis of CD34^+^ cells from the BM of HL patients isolated at diagnosis (*n* = 2) and αPD-1-treated (nivolumab; *n* = 2) HL patients (*p*-value < 0.05). **(F)** Pathway analysis of DEGs of CD34^+^ BM cells isolated from untreated and αPD-1-treated HL patients. *n* = biologically independent mouse and human samples. Heatmaps are normalized log2(CPM); counts per million.

To gain insights into the molecular mechanisms underlying the αPD-L1-mediated expansion and differentiation of HSPCs in tumor-bearing animals, we first evaluated the proteome of BM sera from aPD-L1-treated and control-treated tumor-inoculated mice. Seventy-nine differentially expressed proteins (DEPs, *p* < 0.05) were identified, and 41 exhibited fold change |FC| ≥1.5 (7 upregulated and 34 downregulated in αPD-L1 relative to control; **Supplementary Figure 5A**). To this end, proteins related to hematopoiesis (i.e., *Kars*, *Serpina1c*), stress (*Stip1*, *Stk4*, *Gmps*), and inflammation (*Cndp2*, *Map2k1*, *Cfp*, *Sik2*) were significantly upregulated in the sera from αPD-L1-treated compared to control mice (**Supplementary Figure 5A**). Interestingly, the innate immune receptor melanoma differentiation-associated protein 5 (MDA5; encoded by *Ifih1*), which drives hematopoietic regeneration (27, 28), was exclusively present in the sera from αPD-L1-treated mice, while TGF-β signaling, which has been linked to HSC quiescence (29), was downregulated in αPD-L1-treated mice (**Supplementary Figure 5A**). In support, Gene Ontology analysis (GO) (**Supplementary Figure 5B**), Gene Set Enrichment Analysis (GSEA) (**Supplementary Figure 5C**), and IPA (**Supplementary Figure 5D**) pointed to enhanced inflammation-induced pathways (GO: “TNF signaling pathway” and “interleukin-1 family signaling”, IPA: “LXR/RXR activation” and “NRF2-mediated oxidative stress response,” GSEA: “inflammatory response”), cell cycle (GO: “regulation of cell cycle G1/S phase,” IPA: “HIF1a signaling pathway”), and metabolic reprogramming (GO: “metabolism of nucleotides,” IPA: “glycolysis,” “integrins”, and “iron homeostasis signaling pathway”) in the αPD-L1-treated group of mice. Transcriptomic analysis of HSPCs isolated from B16.F10 melanoma-bearing mice either αPD-L1- or control-treated revealed 59 DEGs (45 upregulated and 13 downregulated in αPD-L1 relative to control, |FC| ≥1.5, FDR <0.05; **Supplementary Table 1**). Among these, genes associated with stress response and inflammation (*Dusp1*, *Fos*, *Zfp36*, *Hspa5*, *Ier2*) were significantly upregulated in HSPCs from αPD-L1-treated compared to control mice (**Figure 6A**). Importantly, genes that regulate HSPC proliferation (*Klf4*, *Pf4*, *Cd69*, *Egr1*) and differentiation (*Klf2*, *Fosb*, *Jun*, *Klf6*) were also upregulated in αPDL-1-treated mice (**Figure 6A**). This was also evident upon pathway analysis of DEGs which showed enrichment in “response to stress pathways,” “response to cytokine,” and “inflammation” (**Figure 6B**) in the αPD-L1-treated group. Supporting these results, GSEA pointed to positive enrichment of pathways such as “negative regulation of myeloid cell differentiation” (NES 1.44, FDR 0.18), “hematopoietic stem cell differentiation,” and “TNF-α signaling via NF-kB” (**Figure 6C**) in HSPCs from αPD-L1-treated compared to control animals. Finally, through IPA, inflammatory-related biological functions such as “S100 signaling,” “dendritic cell maturation,” and “alternative macrophage activation” were predicted to be more active in HSPCs from αPD-L1-treated tumor-bearing animals (**Figure 6D**). Furthermore, transcriptomic analysis on HSPCs isolated from αPD-L1-treated and control MB49-inoculated animals (|FC| > 1.5, FDR < 0.05) revealed 76 DEGs (29 upregulated and 47 downregulated in αPD-L1 relative to control; **Supplementary Table 2**). Interestingly, genes associated with neutrophil development (Camp, Mmp9, Ltf, Lrg1) and maturation (S100A, S100B, Retnlg, Cd177, Wfdc21), myeloid differentiation (Clec5a, Lcn2, Ngp, Lmna), and monocyte activity (*Irf2bp2*) were significantly downregulated in the αPD-L1-treated group, whereas genes involved in cell self-renewal (Egln1, Thbs1) were significantly upregulated (**Supplementary Figure 5E**). GO analysis showed significant enrichment of terms such as “response to stress,” “myeloid differentiation,” and “inflammatory response” in HSPCs isolated from αPD-L1-treated mice compared to control animals (**Supplementary Figure 5F**). Additionally, GSEA analysis indicated a negative enrichment in “myeloid cell differentiation” and a positive enrichment of “interferon alpha response” (**Supplementary Figure 5G**) in HSPCs treated with αPD-L1 compared to control conditions. Collectively, these results demonstrate that αPD-L1 immunotherapy causes transcriptomic reprogramming in HSPCs in the BM of both immunogenic and non-immunogenic cancer cell-inoculated animals.

Interestingly, transcriptomic analysis of CD34^+^ cells from the BM of αPD-1-treated individuals with HL revealed 612 DEGs (**Supplementary Table 3**), of which 202 were upregulated and 410 were downregulated (*p*-value < 0.05) compared to CD34^+^ cells from samples from HL patients isolated at diagnosis. Specifically, genes associated with HSC expansion (*CXCL8*, *DSG2*, *ZBTβ4*, *MYCτ1*, *PROM1*, *DOCK1*), self-renewal (*RORA*, *PLAG1*, *SPARC*, *SEL1L*, *PRDM16*, *PBX1*, *GAB2*, *CABLES1*), proliferation (*EGR1*, *KAZALD1*, *ATF3*), and differentiation (*JUN*, *COL5A1*) were significantly downregulated in CD34^+^ cells from αPD-1-treated individuals compared to untreated (**Figure 6E**). Notably, genes that participate in HSC differentiation toward the myeloid cell lineage (*HOXA3*, *NAP1L3*, *RUNX3*, *RGS18*, *EVI2B*, *FZD6*, *CCL3*) were also downregulated in the αPD-1-treated group, while upregulation of *POU2AF1*, *RAG2*, *CD19*, *CD79A*, and *CD79B* that pointed to skewing toward the development of lymphoid progenitors was evident in CD34^+^ cells from αPD-1-treated individuals (**Figure 6E**). GO further supported these findings with lymphocyte and myeloid differentiation pathways to be highly enriched as well as the response to stimulus pathway in treated patients, in accordance with the αPD-L1-treated mouse data (**Figure 6F**). Overall, the findings presented here show that αPD-L1 promotes a transcriptomic reprogramming of HSPCs underlined by inflammatory-related processes.

### αPD-L1 immunotherapy modulates the myelopoiesis potential of HSPCs during cancer

**Figure 7:**
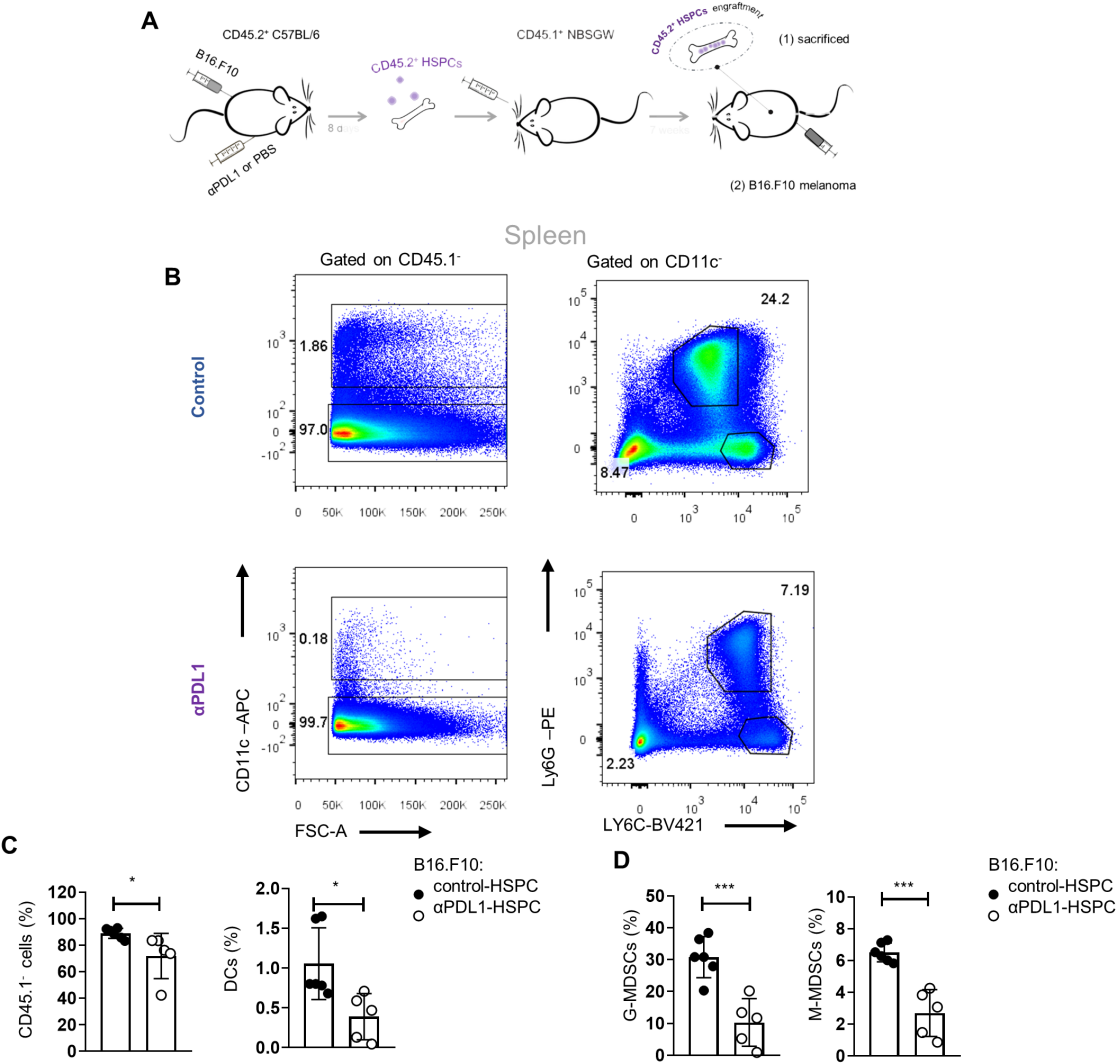
αPD-L1 immunotherapy rewires cancer emergency myelopoiesis. **(A)** Experimental scheme: HSPCs (Lin^−^Sca1^+^cKit^+^) isolated 8 days from B16.F10 tumor-bearing C57BL/6 (CD45.2^+^CD45.1^−^) mice treated with PBS (control-HSPC) or αPD-L1 (αPD-L1-HSPC) and then adoptively transferred to NBSGW mice (CD45.2^−^CD45.1^+^). Following 7 weeks of HSPC engraftment, the recipient mice were either sacrificed and analyzed [**A**(1)] or inoculated with B16.F10 and sacrificed after 17 days of tumor development [(**A**(2)]. **(B)** Numbers denote the percentages of gated populations. Representative FACS plots of splenic DC cells, G-MDSCs, and M-MDSCs isolated from melanoma-bearing NBSGW [as in (**A**(2)]. **(C, D)** Frequencies in total cells of splenic CD45.1^−^ cells, DCs (**C**; *n* = 6 control-HSPC, *n* = 5 αPD-L1-HSPC), G-MDSCs, and M-MDSCs (**D**; *n* = 6 control-HSPC, *n* = 5 αPD-L1-HSPC) in NBSGW mice inoculated with B16.F10 and sacrificed after 17 days [as in **A**(2)]. Data from two combined independent experiments. *p* < 0.05*, *p* < 0.01**, *p* < 0.001***, *p* < 0.0001****. If not stated otherwise, unpaired two-tailed *t*-tests were performed. Means and SEM are depicted in all bar plots. *n* = biologically independent mouse samples.

We showed that αPD-L1 immunotherapy promotes the exit of the HSPC compartment from quiescence and induces their transcriptomic rewiring, raising the possibility of altered cancer myelopoiesis. To provide direct evidence for an αPD-L1-mediated altered myelopoiesis *in vivo*, we performed a transplantation experiment, as depicted in **Figure 7A**, where BM CD45.2^+^ HPSCs isolated from aPD-L1- or PBS-treated β16.β10 tumor-bearing mice were transplanted into CD45.1^+^NOD.Cg-*Kit^W-41J^Tyr* ^+^ *Prkdc^scid^Il2rg^tm1Wjl^*/ThomJ (NBSGW) hosts, which support the multilineage engraftment of hematopoietic cells. The analysis of the myeloid population composition of the spleen 7 weeks after transplantations showed successful engraftment and comparable myelopoiesis potential between both groups [**Figure 7A**(1) and **Supplementary Figures 6A–C**]. Importantly, inoculation of mice with B16.F10 tumor cells [**Figure 7A**(2)] demonstrated a rewiring of the myelopoiesis potential of HSPCs, derived from αPD-L1-treated donor mice, as shown by the decreased frequencies of myeloid cells, including DCs, and both MDSC subsets (**Figures 7B–D**). Collectively, our findings demonstrate that αPD-L1 immunotherapy alters the myelopoiesis program of HSPCs, altering their susceptibility to cancer-induced myelopoiesis.

## Discussion

Blockade of the PD-L1/PD-1 axis constitutes a highly promising therapy in a broad spectrum of solid tumors, which however elicits durable antitumor responses and long-term remissions only in a small subset of patients (30, 31). Despite major research efforts, current biomarkers of response, such as tumor mutational burden (TMB), PD-L1 expression, T-cell infiltration, and IFN-γ expression (32), demonstrate very low prediction power. For example, a recent meta-analysis showed that high TMB predicts responsiveness to αPD-L1 only in 25% of patients with various types of cancer (33). Similarly, the lack of PD-L1 expression cannot reliably exclude responses to αPD-L1 or αPD-1 ICβ (34). Another major challenge is that clinical responses to PD-1/PD-L1 blockade are often accompanied by the development of adverse events resembling autoimmune reactions (35). Therefore, to understand the resistance mechanisms to immunotherapy and to design rational immunotherapies in cancer with diminished adverse events, it is necessary to delineate the unappreciated mechanisms of PD-1/PD-L1 axis targeted therapy. In this direction, herein, we demonstrate that αPD-L1 immunotherapy targets the HSPC compartment in the BM and rewires the cancer emergency myelopoiesis.

Since PD-1 engagement to PD-L1 imprints on T-cell function to maintain tolerance (36), it was reasonable to focus on T-cell-mediated antitumor immune responses as a potential mode of action of PD-1/PD-L1 targeting. However, recent studies demonstrate a broader expression of PD-1 and PD-L1, which adds a level of complexity to the so far proposed mechanisms. For example, PD-1 is also expressed by monocytic lineage cells, whereas PD-L1 is expressed by CD8 T cells, fibroblasts, and endothelial cells (7, 37–39). Our findings demonstrate that HSPCs express PD-L1 at a steady state and that it is upregulated depending on tumor immunogenicity. Although previous studies have shown the expression of PD-L1 by HSPCs (40–42), in this study, we demonstrate that not only are they targeted by αPD-L1 immunotherapy but also that this interaction alters their fate and differentiation program. Specifically, αPD-L1 immunotherapy promotes the expansion of HSPCs and induces their exit from quiescence, which is further supported by experiments with genetic ablation of the PD-1/PD-L1 axis. Molecularly, we showed that inflammatory signaling is activated by treatment with αPD-L1. Indeed, inflammatory signaling like IFN, S100A, TLR, IL-1, and TNF is well established to promote activation and differentiation (43–47). LSK cells constitute a heterogeneous cell population, and transcriptomic differences may also reflect differences in the abundance of the diverse cell subsets within the compartment. Therefore, future studies may aim to identify which cell subset(s) are responsible for the inflammatory reprogramming observed upon αPD-L1 treatment in cancer-inoculated animals. Functionally, this is translated by an altered cancer-associated emergency myelopoiesis as shown by the reduced myeloid cell frequency upon transplantation of αPD-L1-treated HSPCs.

Tumor-associated myeloid cells constitute a heterogeneous population of cells that dictate the fate of tumor development. Tumor-associated macrophages (TAMs) and neutrophils (TANs), MDSCs, and DCs are the most abundant cells of myeloid origin in the TME (48) and mainly exert a tumor-promoting function. It is established that the majority of those cells originate from the BM through emergency myelopoiesis, which is directed by the nature of tumor cells (49). The unique characteristic of tumor-associated emergency myelopoiesis is the emergence of immature myeloid cells with intense immunosuppressive activities (49). Although in our study MDSCs are reduced in the periphery of αPD-L1-treated mice, it may be possible that they are retained in the BM. Our preliminary results do not support this hypothesis since similar frequencies of myeloid cells were observed in the BM of control and αPD-L1-treated mice 8 days following B16.F10 melanoma inoculation (data not shown). Nevertheless, a kinetic experiment is required to directly address this hypothesis.

Extensive research endeavors are focused on the reprogramming of cancer-associated emergency myelopoiesis to improve immunological performances against tumors. This has proven challenging due to myeloid cell heterogeneity and plasticity. Importantly, targeting strategies are focused on “terminally” differentiated myeloid cells (50), while efforts to interfere with myelopoiesis in the BM are limited. Interfering with cancer-associated myelopoiesis has been shown to be beneficial for host-promoting tumor regression in tumor-bearing mice. For example, transcriptomic and epigenetic rewiring of myelopoiesis induced by B-glucans resulted in the generation of granulocytes with antitumor activities, and this effect was transmissible by BM transplantation to naive recipient mice (51). In line with this, our results showed that transplantation of αPD-L1-treated HSPCs resulted in the reprogramming of tumor-induced myelopoiesis, as evidenced by the reduced potential of HSPCs from mice treated with αPD-L1 to generate MDSCs in tumor-bearing recipient mice. Whether αPD-L1 acts intrinsically on HSPCs or extrinsic mechanisms also participate in promoting HSPC reprogramming remains to be investigated. Antibodies against PD-L1 have been shown to induce reverse signaling upon binding to tumor cells (52) but also to DCs (53) and macrophages (13). Accordingly, we show that αPD-L1 treatment targets HSPCs in the BM, raising the possibility of a reverse signaling operation in the rewiring of emergency myelopoiesis. However, since systemic administration of αPD-L1 is known to interfere with adaptive immune responses, the contribution of extrinsic mechanisms, such as the release of inflammatory cytokines and soluble factors, in HSPC reprogramming cannot be excluded. In contrast, genetic or pharmacological inhibition of PD-L1 was shown to suppress the development of inflammatory macrophage in a yolk sac organoid culture (54). However, it is unlikely that this *in-vitro* system provides all the necessary signals to mimic the *in-vivo* development of macrophages.

From a mechanistic point of view, our data support the potential role of lymphocytes in the rewiring of tumor emergency myelopoiesis since αPD-L1 treatment of tumor-bearing *RAG1^−/−^* animals failed to induce the expansion of HSPCs in the BM. Recent evidence shows that Tregs in the BM are essential in regulating HSC quiescence, while specific ablation of BM Treg cells leads to the expansion of HSCs and colony formation *in vitro* (55). Considering that Treg cells express high levels of PD-1, combined with our results showing that interruption of the PD-L1/PD-1 axis leads to expansion of HSPCs in tumor-bearing mice, it is plausible that Treg cells may co-ordinate the cancer-associated emergency myelopoiesis through the PD-1 axis. Although the crosstalk of T-cell subsets with HSCs has been previously reported (56, 57), whether this is supported by the PD-1/PD-L1 axis remains to be determined. Interestingly, another study showed that myeloid cell-specific ablation of PD-1 altered the emergency myelopoiesis, with myeloid progenitors such as CMPs and GMPs expressing high levels of PD-1 (37). Thus, the involvement of myeloid cells or stromal cells expressing PD-1 in shaping HSPC quiescence cannot be excluded. Of interest, in addition to PD-1, an interaction of PD-L1 with CD80 has been reported in mouse models (58). Both activated T cells (59) and myeloid cells express CD80 (60, 61); therefore, better characterization of the mechanisms that govern PD-L1-mediated HSPC quiescence is required.

Treatment with αPD-L1 caused transcriptomic reprogramming of HSPCs with the upregulation of inflammatory pathways. Indeed, these findings imply that the nature of myeloid cells that exit the BM upon αPD-L1 treatment may possess an antitumor/inflammatory activity rather than a protumorogenic/suppressive function. Accordingly, ablation of the PD-L1/PD-1 axis changed the balance of myeloid cells exiting the BM with reduced MDSCs and increased effector myeloid cell frequencies (37). Thus, our findings may also hold important implications in the emergence of irAEs observed in patients responding to ICB immunotherapy. Although activation of effector CD4^+^ and CD8^+^ T cells (62, 63) as well as disturbances in Treg cells (64, 65) are implicated in irAE development, inflammatory monocytes are expected to contribute in a direct way through the secretion of proinflammatory cytokines and chemokines or indirectly via antigen processing and presentation. However, the role of emergency myelopoiesis in irAEs has not been examined.

To conclude, our findings reveal the targeting of the HSPC compartment in the BM by αPD-L1 immunotherapy, which reprograms the cancer-associated emergency myelopoiesis. Considering that the PD-L1/PD-1 axis constitutes a major therapeutic target in solid tumors and hematologic malignancies, our data provide significant insights into therapy resistance mechanisms and the development of immune adverse events. Finally, the results described here place the BM microenvironment as a target of ICB immunotherapy for the future design of rational immunotherapy for cancer treatment.

## Data Availability

The data presented in the study are deposited in the following repositories: Proteomics raw data files are available on the MassIVE repository (identifier: MSV000095541) and on the ProteomeXchange repository (identifier: PXD054676). Human genomic data is deposited in the European Genome-phenome Archive (EGA) under accession number EGAS00001007873. Additionally, the mouse RNA-seq data have been deposited in the NCBI SRA repository under BioProject accession number PRJNA1155705.
