# Supplementary figures and images for: PD-L1 blockade immunotherapy rewires cancer-induced emergency myelopoiesis
Source: Front Immunol. 2024 Oct 11;15:1386838. doi: 10.3389/fimmu.2024.1386838 (PMC11502414; doi:10.3389/fimmu.2024.1386838)

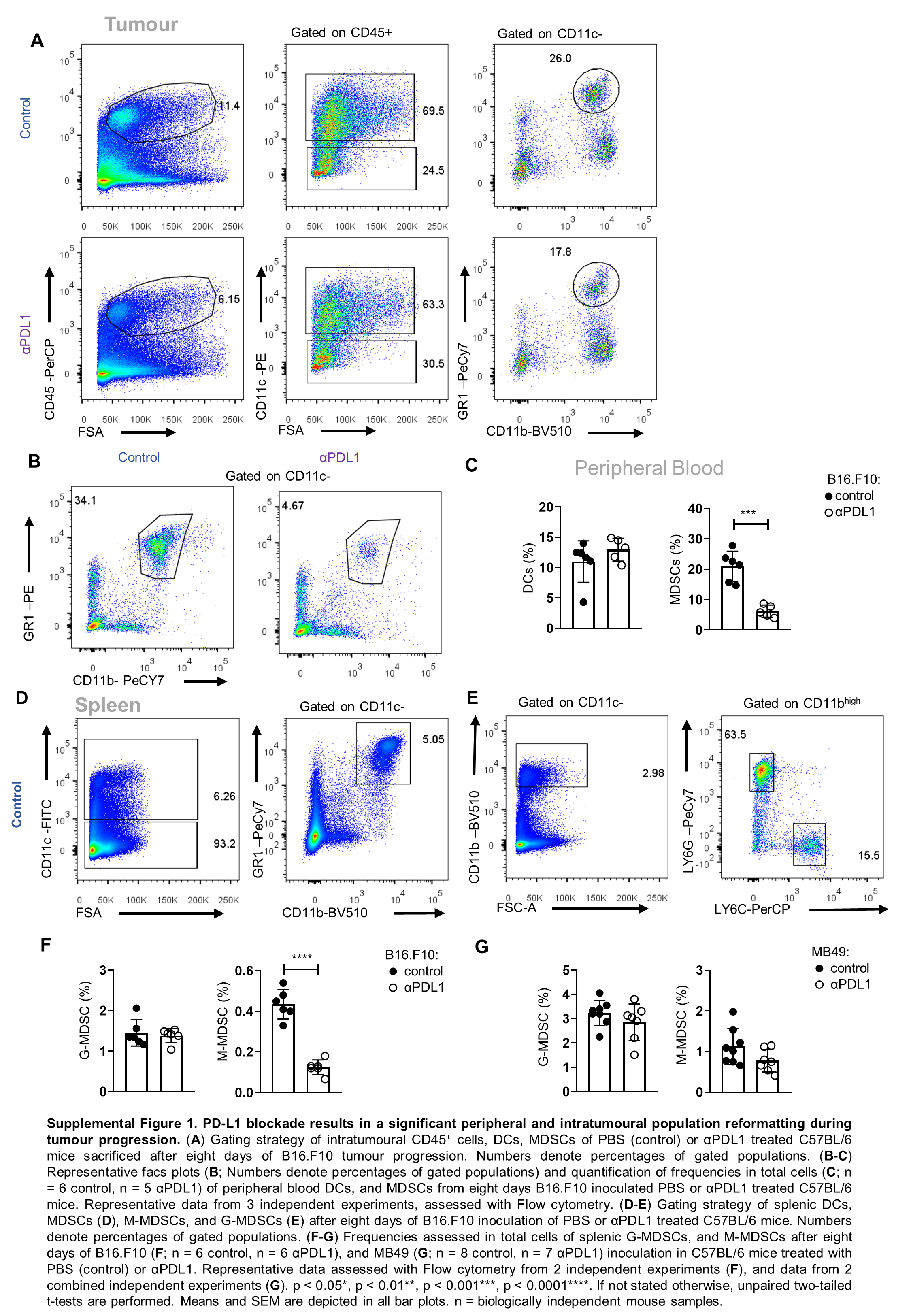

Supplement: Supplementary file 1 [file Image1.tif]

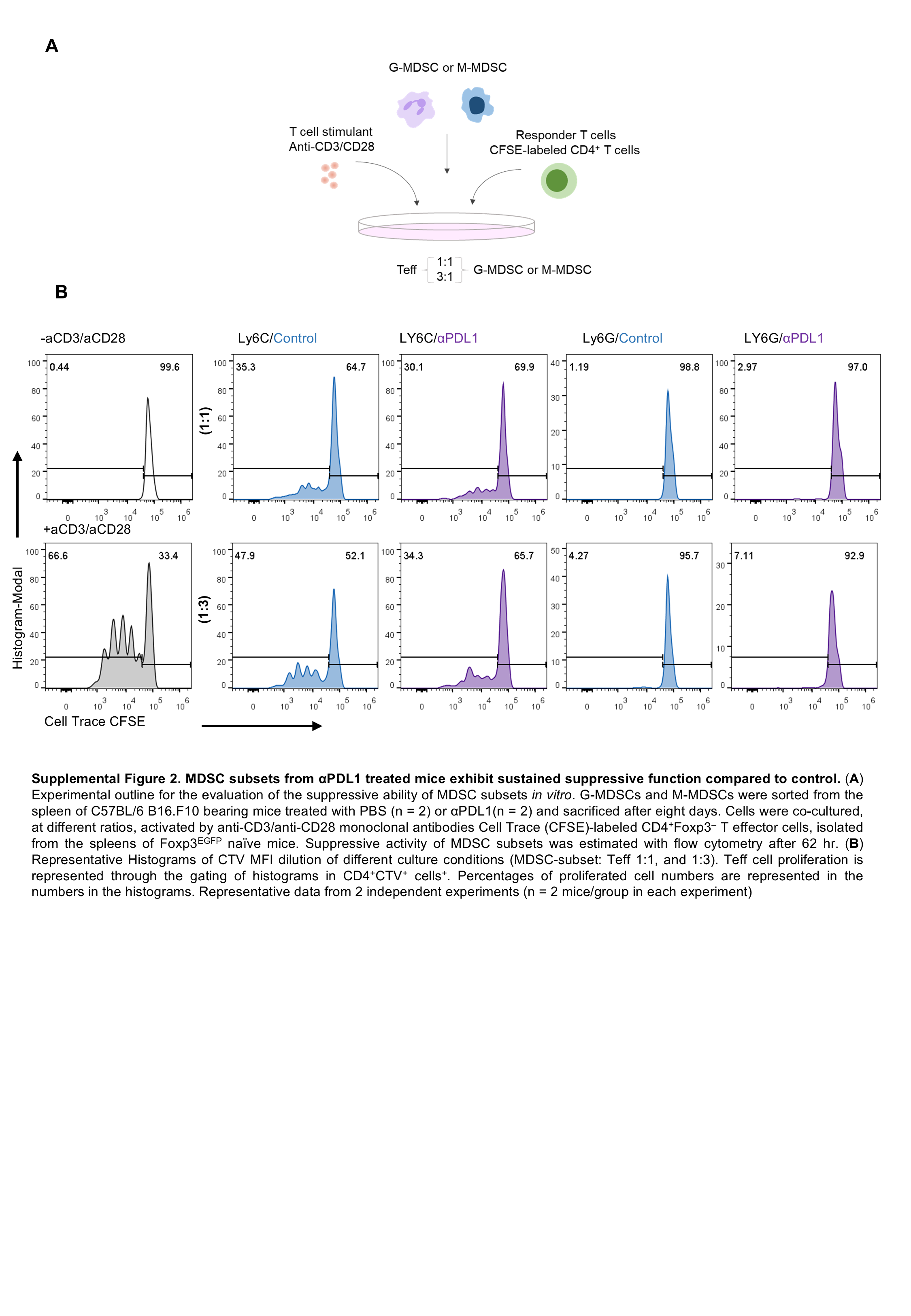

Supplement: Supplementary file 2 [file Image2.tif]

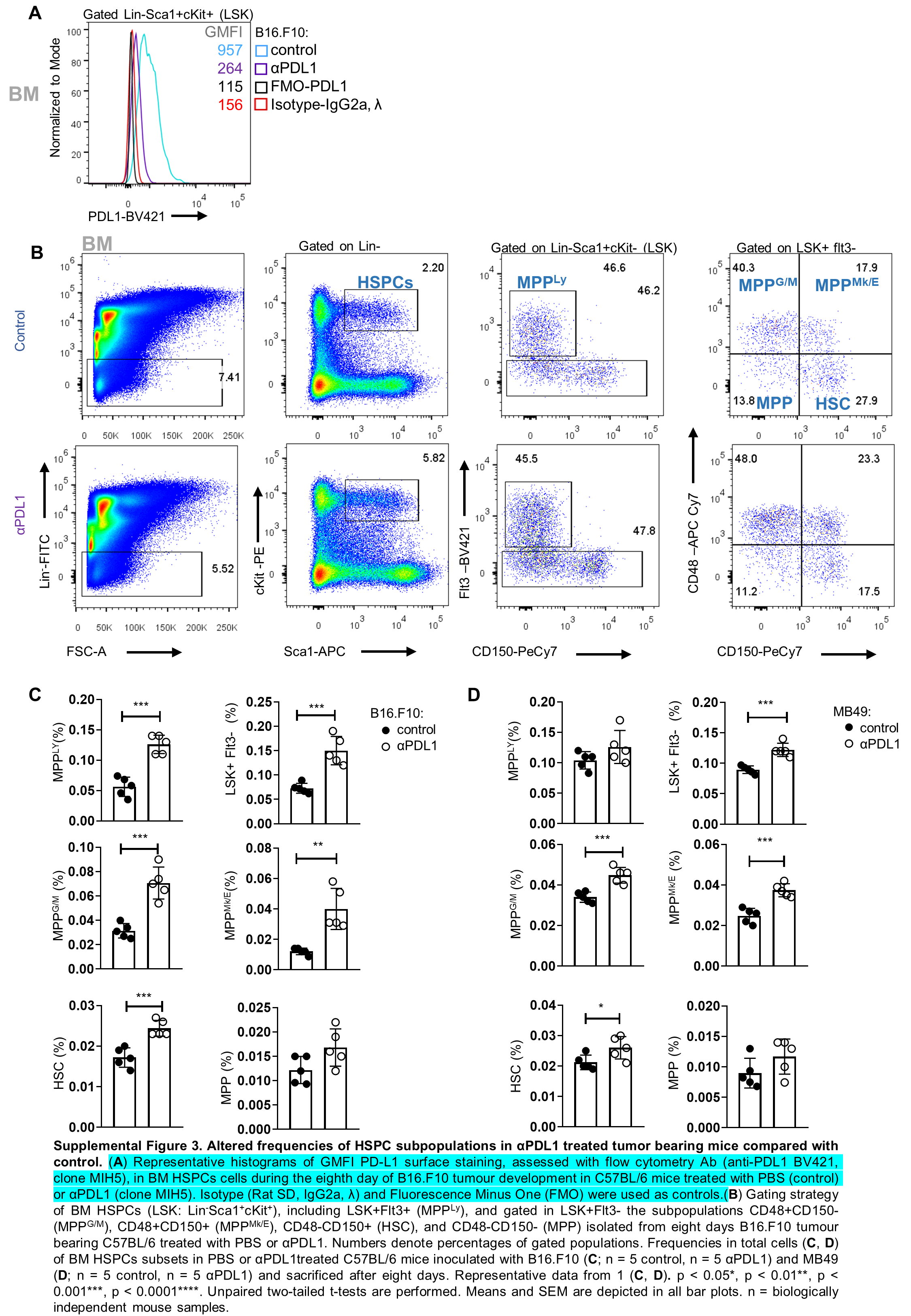

Supplement: Supplementary file 3 [file Image3.tif]

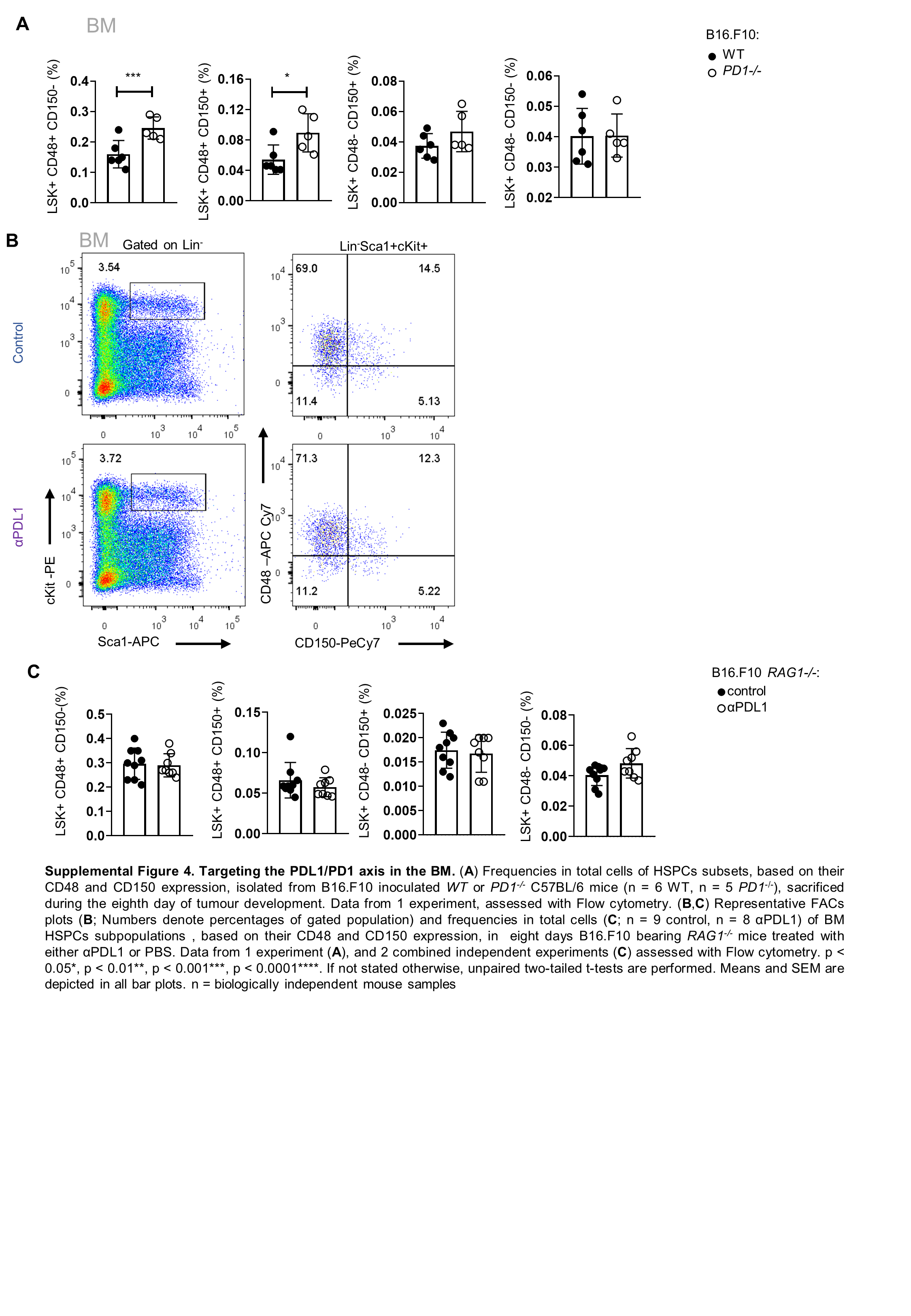

Supplement: Supplementary file 4 [file Image4.tif]

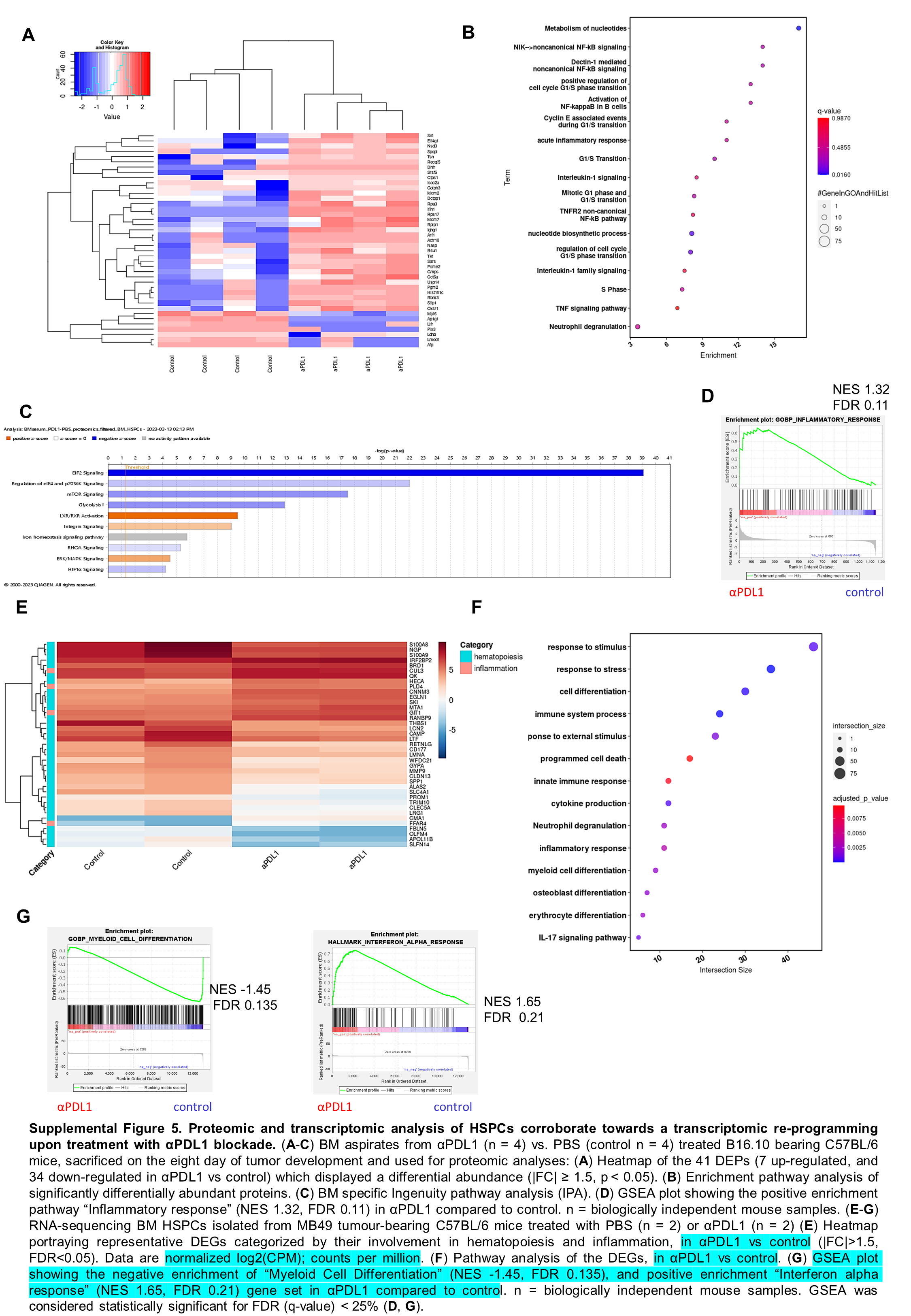

Supplement: Supplementary file 5 [file Image5.tif]

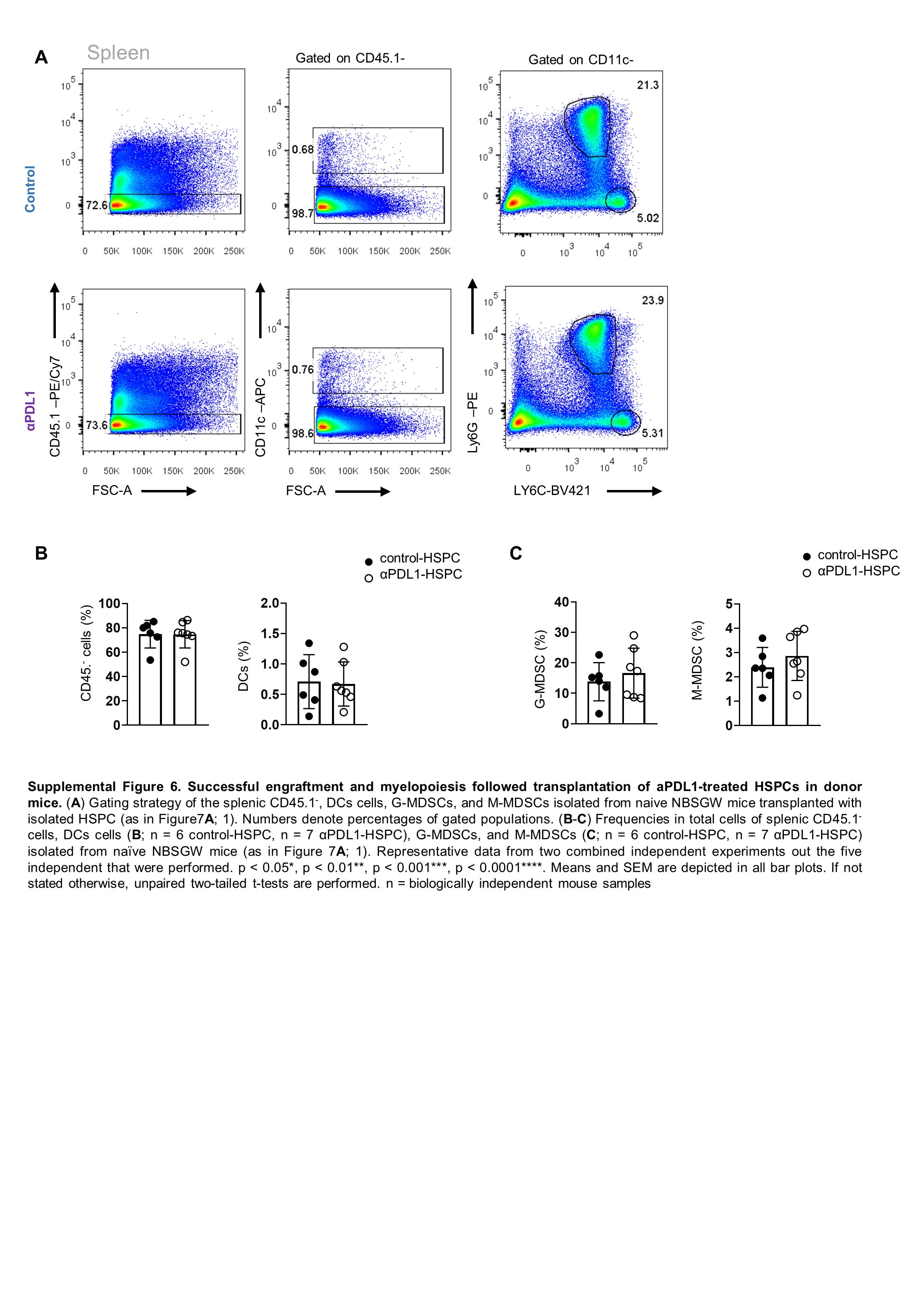

Supplement: Supplementary file 6 [file Image6.tif]
